# LMP1-mediated glycolysis induces myeloid-derived suppressor cell expansion in nasopharyngeal carcinoma

**DOI:** 10.1371/journal.ppat.1006503

**Published:** 2017-07-21

**Authors:** Ting-Ting Cai, Shu-Biao Ye, Yi-Na Liu, Jia He, Qiu-Yan Chen, Hai-Qiang Mai, Chuan-Xia Zhang, Jun Cui, Xiao-Shi Zhang, Pierre Busson, Yi-Xin Zeng, Jiang Li

**Affiliations:** 1 Collaborative Innovation Center for Cancer Medicine, State Key Laboratory of Oncology in South China, Guangzhou, China; 2 Guangdong Provincial Key Laboratory of Colorectal and Pelvic Floor Diseases, The Sixth Affiliated Hospital, Sun Yat-sen University, Guangzhou, China; 3 Department of Biotherapy, Sun Yat-sen University Cancer Center, Guangzhou, China; 4 Department of Nasopharyngeal Carcinoma, Sun Yat-sen University Cancer Center, Guangzhou, China; 5 Key Laboratory of Gene Engineering of the Ministry of Education, State Key Laboratory of Biocontrol, College of Life Sciences, Sun Yat-sen University, Guangzhou, China; 6 Université Paris-Sud-11, CNRS-UMR 8126 and Institut de Cancérologie Gustave Roussy, 39 rue Camille Desmoulins, Villejuif, France; Northwestern University, UNITED STATES

## Abstract

Myeloid-derived suppressor cells (MDSCs) are expanded in tumor microenvironments, including that of Epstein–Barr virus (EBV)-associated nasopharyngeal carcinoma (NPC). The link between MDSC expansion and EBV infection in NPC is unclear. Here, we show that EBV latent membrane protein 1 (LMP1) promotes MDSC expansion in the tumor microenvironment by promoting extra-mitochondrial glycolysis in malignant cells, which is a scenario for immune escape initially suggested by the frequent, concomitant detection of abundant LMP1, glucose transporter 1 (GLUT1) and CD33^+^ MDSCs in tumor sections. The full process has been reconstituted *in vitro*. LMP1 promotes the expression of multiple glycolytic genes, including GLUT1. This metabolic reprogramming results in increased expression of the Nod-like receptor family protein 3 (NLRP3) inflammasome, COX-2 and P-p65 and, consequently, increased production of IL-1β, IL-6 and GM-CSF. Finally, these changes in the environment of malignant cells result in enhanced NPC-derived MDSC induction. One key step is the physical interaction of LMP1 with GLUT1 to stabilize the GLUT1 protein by blocking its K48-ubiquitination and p62-dependent autolysosomal degradation. This work indicates that LMP1-mediated glycolysis regulates IL-1β, IL-6 and GM-CSF production through the NLRP3 inflammasome, COX-2 and P-p65 signaling pathways to enhance tumor-associated MDSC expansion, which leads to tumor immunosuppression in NPC.

## Author summary

The expression of the Epstein-Barr virus (EBV) oncogenic protein denoted latent membrane protein 1 (LMP1) varies in patients with NPC and is linked to tumorigenesis and tumor immunosuppression, but the molecular mechanism through which LMP1 leads to tumor immune escape remains unknown. Work to date suggests that the expansion of tumor-associated myeloid-derived suppressor cells (MDSCs) is the main cause of tumor immunosuppression such as that found in NPC. Here, we found that tumor LMP1 expression is correlated with glucose transporter 1 (GLUT1) levels, CD33^+^ MDSC number and unfavorable survival in patients with NPC. Based on the results of our *in vitro* analysis, LMP1 promotes GLUT1-dependent glycolysis in NPC cells, resulting in activation of the Nod-like receptor family protein 3 (NLRP3) inflammasome, COX-2 and P-p65 signaling pathways and subsequently increased IL-1β, IL-6 and GM-CSF production. Importantly, LMP1 interacts with GLUT1 to stabilize the GLUT1 protein by disrupting its K48-linked ubiquitination and autolysosomal degradation in a p62-dependent manner and up-regulating the GLUT1 mRNA and protein levels by inducing p65 activation. Therefore, we determined that GLUT1-dependent glycolysis is required for tumor-induced MDSC differentiation and that this process is associated with LMP1 expression. Based on our findings, LMP1-mediated glycolysis is a key process involved in controlling tumor immunosuppression and directly contributes to oncogenesis.

## Introduction

Ninety-five percent of nasopharyngeal carcinoma (NPC) cases in South China are of the undifferentiated histological type (WHO type III), which is associated with Epstein-Barr virus (EBV) infection. A type II latency program, which includes the expression of latent membrane proteins 1 and 2 (LMP1 and LMP2), EBV nuclear antigen 1 (EBNA1) and EBV-encoded RNAs (EBERs), is often operating in EBV-infected NPC cells [1]. Among these latent type II antigens, LMP1 has been identified as an oncoprotein and is essential for the maintenance of latent infection and EBV-mediated malignant transformation [2, 3]. It enhances the production of angiogenic factors and the *in vivo* formation of the neovasculature for rapid tumor cell invasion and metastasis. LMP1 also has a strong impact on genes linked to inflammation and antigen presentation. These modifications might have opposite consequences. On the one hand, they might facilitate tumor progression; on the other hand, they can favor immune exposure and tumor rejection [2, 4, 5]. Overall, the role of LMP1 in the interaction of NPC tumors with the immune system requires additional investigations that consider all types of immune cells, including myeloid-derived suppressor cells (MDSCs).

Oxidative phosphorylation and extra-mitochondrial glycolysis are the two major energy-producing pathways in a cell [6]. In cancers, most cells exhibit increased rates of extra-mitochondrial glycolysis and use this metabolic pathway for ATP synthesis instead of oxidative phosphorylation, even in the presence of oxygen, which is a process known as the Warburg effect [7]. The high rate of extra-mitochondrial glycolysis not only causes malignant cells to be more prone to resist hypoxia but also contributes to cell proliferation and survival by affecting signaling pathways and enhancing the production of various macromolecules, such as proteins, nucleic acids, and lipids [8, 9]. Recent studies have suggested that EBV LMP1 may mediate energy metabolism reprogramming in EBV-infected cancer cells, including alterations in aerobic glycolysis, by activating the expression of specific metabolic enzymes, such as hexokinase 2 (HK2) [10–12]. Several reports have shown that this metabolic reprogramming has major consequences for oncogenesis and responses to treatment [13]. Its impact on the tumor microenvironment, particularly on the interaction between malignant and immune cells, also deserves attention. For example, a link between metabolic reprogramming in malignant cells and the expansion of intra-tumor MDSCs has been reported [14]. MDSCs are key regulatory cells that have a physiological role in the control of inflammation [15]. They are also known to favor tumor immune escape [16–21]. According to many reports, the MDSC subset is expanded in the tumor microenvironment in a wide range of malignancies, including NPC.

The aim of this study was to investigate the role of metabolic reprogramming as a missing link between LMP1 expression in malignant cells and the accumulation of MDSCs in the tumor microenvironment. An initial clue supporting this hypothesis was provided by immunohistochemical (IHC) analyses of tumor sections; the abundance of CD33^+^ MDSCs was correlated to the level of LMP1 and glucose transporter 1 (GLUT1) expression in malignant epithelial cells. The next step was to confirm that aerobic extra-mitochondrial glycolysis was increased by LMP1 through up-regulation of several glycolytic genes, including GLUT1. Then, we showed that MDSC expansion *in vitro* was facilitated by the expression of exogenous LMP1 in neighboring NPC cells. Mechanistic analyses indicated that LMP1-mediated glycolysis was GLUT1-dependent. The increase in extra-mitochondrial glycolysis resulted in enhanced COX-2 expression and the phosphorylation of p65 in the nuclear factor-κB (NF-κB) signaling pathway. Increased extra-mitochondrial glycolysis also triggered Nod-like receptor family protein 3 (NLRP3) inflammasome activity, with increasing NLRP3 expression and self-cleavage of caspase-1 and IL-1β. The activation of the above-mentioned signaling pathways resulted in greater levels of extracellular IL-1β, IL-6 and GM-CSF release. Moreover, we determined that GLUT1-dependent glycolysis was required for MDSC differentiation, which was associated with LMP1 expression. Importantly, we observed that LMP1 interacted with GLUT1 to stabilize the GLUT1 protein by disrupting its K48-linked ubiquitination and autolysosomal degradation in a p62-dependent manner in addition to up-regulating GLUT1 mRNA and protein expression through p65 activation. The authenticity of this article has been validated by uploading the key raw data onto the Research Data Deposit (RDD) public platform (www.researchdata.org.cn), with the RDD approval number RDDB2017000052

## Results

### Correlation of LMP1 and GLUT1 expression with the abundance of CD33^+^ MDSCs in patients with NPC

**Fig 1:**
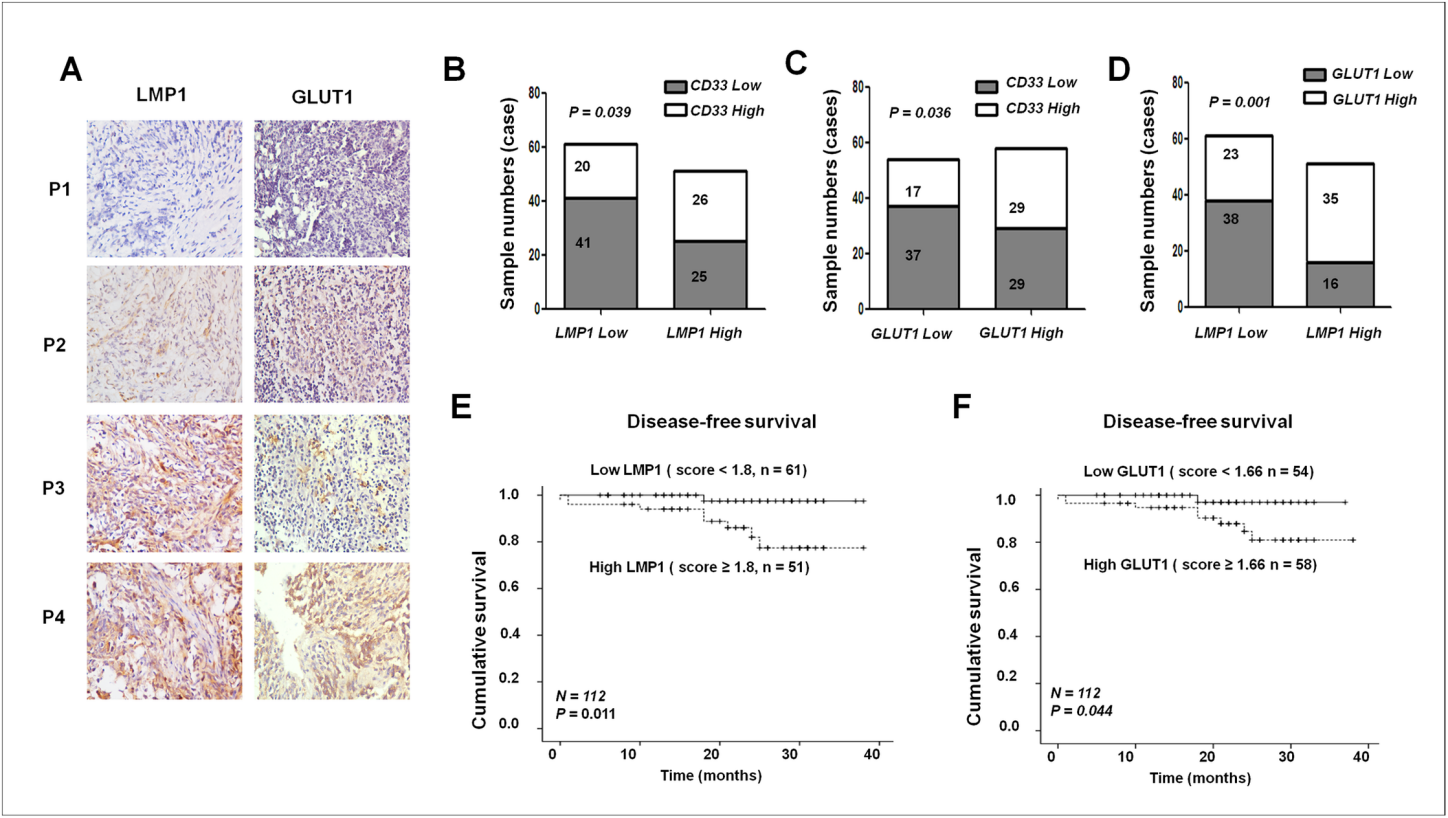
Positive correlations were observed between the abundance of LMP1 and GLUT1 in malignant cells and the number of CD33^+^ MDSCs in NPC tissues. (**A**) Representative IHC staining for LMP1 and GLUT1 in NPC specimens. (**B-D**) Statistical analyses of the correlations between LMP1 expression and CD33^**+**^ MDSC number (**B**), GLUT1 expression and CD33^**+**^ MDSC number (**C**), and LMP1 and GLUT1 expression level (**D**), Pearson’s chi-square test was used to assess statistical significance. (**E-F**) Kaplan-Meier estimates of the progression-free interval were determined based on the median levels of LMP1 (**E**) and GLUT1 (**F**) in the patients with NPC. P < 0.05 was considered statistically significant.

Striking inter- and intra-tumor heterogeneity in LMP1 expression has been observed in NPC tumors [22, 23]. To understand the possible relationship between LMP1 expression in malignant cells, the Warburg effect and immune alterations in the tumor microenvironment, our first approach was based on IHC analyses of tumor tissue sections from 112 patients with NPC. The expression of LMP1 and GLUT1 in malignant cells was assessed in combination with counting the number of infiltrating immune cells, particularly MDSCs. We found a positive correlation between 3 parameters: the abundance of LMP1 proteins in malignant cells, the abundance of GLUT1 proteins in malignant cells and the number of CD33^+^ MDSCs in the tissue sections [18] (Fig 1A–1D, P < 0.05). Importantly, high levels of LMP1 or GLUT1 in tumors were associated with a poorer disease-free survival (DFS) of patients with NPC (Fig 1E and 1F, S2 Table). A high density of tumor-infiltrating CD33^+^ cells was also indicative of a poor prognosis, as shown in our previous study [18].

### LMP1 enhances aerobic glycolysis and the expression of glycolysis-related genes in NPC cells

**Fig 2:**
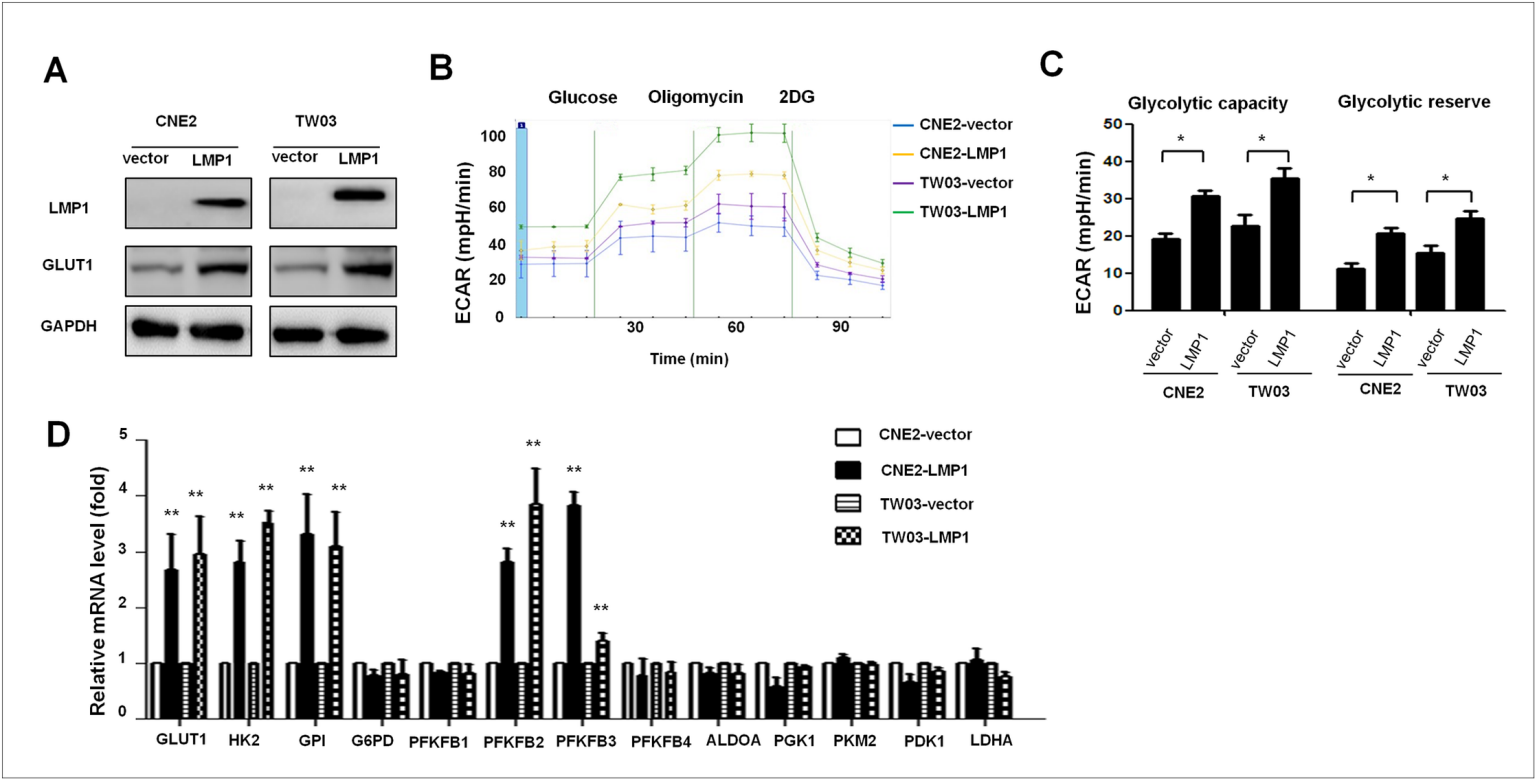
LMP1 increased glycolysis and the expression of glycolysis-related genes in NPC cells. (**A**) Exogenous EBV oncoprotein LMP1 was stably overexpressed in the NPC cell lines CNE2 and TW03 following lenti-LMP1 transfection, and the lenti-vector was included as control. WB showed that the expression of LMP1 and GLUT1 was increased in CNE2-LMP1 and TW03-LMP1 cells compared with that in CNE2-vector and TW03-vector cells, and GAPDH was included as a control. One representative experiment of five independent experiments is shown. (**B-D**) ECAR assay results. CNE2-vector, CNE2-LMP1, TW03-vector and TW03-LMP1 cells were cultured in base DMEM media with no glucose or glutamine. ECAR was assessed after the addition of 10 mM glucose and in response to the metabolic inhibitor oligomycin and the glucose inhibitor 2-DG. The time course and calculations for (**B**) glycolytic capacity and (**C**) statistical analysis of glycolytic capacity and glycolytic reserve are shown. (**D**) The relative mRNA levels of glycolysis-related genes, including GLUT1, HK2, GPI, PFKB1, 2, 3 and 4, ALDOA, PGK1, PKM2 and LDHA, in CNE2-vector, CNE2-LMP1, TW03-vector and TW03-LMP1 cells were measured via real-time qRT-PCR. Statistical results are representative of at least three experiments performed in triplicate. All values are shown as the means ± SEM; * indicates P < 0.05. ** indicates P < 0.01.

To identify a formal link between LMP1 expression and metabolic reprogramming, we investigated the impact of exogenous LMP1 expression on glucose metabolism in the EBV-negative NPC cell line CNE2 and in TW03 cells. We found that GLUT1 expression was induced by LMP1 expression in both cell lines (Fig 2A). We then measured glucose consumption and lactate production based on the extracellular acidification rate (ECAR) using an extracellular metabolic flux analyzer to assess the differences in glycolytic activity among CNE2-LMP1, TW03-LMP1, CNE2-vector and TW03-vector cells, as described in the Methods. CNE2-LMP1 and TW03-LMP1 cells showed significantly increased ECARs following the addition of glucose compared with those of CNE2-vector and TW03-vector cells. These results suggest that in the presence of LMP1, the added glucose was almost entirely degraded in the extra-mitochondrial glycolytic pathway, which resulted in the abundant release of lactic acid, thus providing evidence of a high glycolytic capacity. Then, oligomycin was added to block mitochondrial ATP production and induce maximal rates of extra-mitochondrial glycolysis. Importantly, CNE2-LMP1 and TW03-LMP1 cells each showed robust increases in their ECAR following oligomycin treatment, which was in contrast to the minimal changes observed in the control cells. These observations suggest that in the presence of LMP1, the shortage of ATP resulting from oligomycin treatment was compensated for by more intense extra-mitochondrial glycolysis and a more abundant extra-mitochondrial release of lactate, which provides evidence of a high glycolytic reserve. Finally, as expected, ECARs were dramatically reduced in all cell lines following the administration of the glycolytic pathway inhibitor 2-deoxy-D-glucose (2-DG) (Fig 2B). In conclusion, the glycolytic capacity and glycolytic reserve were both substantially increased in CNE2-LMP1 and TW03-LMP1 cells in comparison with these parameters in control cells (Fig 2C). Consistently, we found major changes in the expression of several genes encoding the transmembrane carrier GLUT1 and key enzymes involved in extra-mitochondrial glycolysis. The expression levels of GLUT1, HK2, glucose phosphate isomerase (GPI), and 6-phosphofructo-2-kinase/fructose 2 and 3 (PFKFB2 and 3) were dramatically elevated in CNE2-LMP1 and TW03-LMP1 cells compared with the levels in control cells (Fig 2D).

### LMP1 promotes the expression of MDSC-related molecules and cytokines leading to the induction of NPC-associated MDSCs *in vitro*

**Fig 3:**
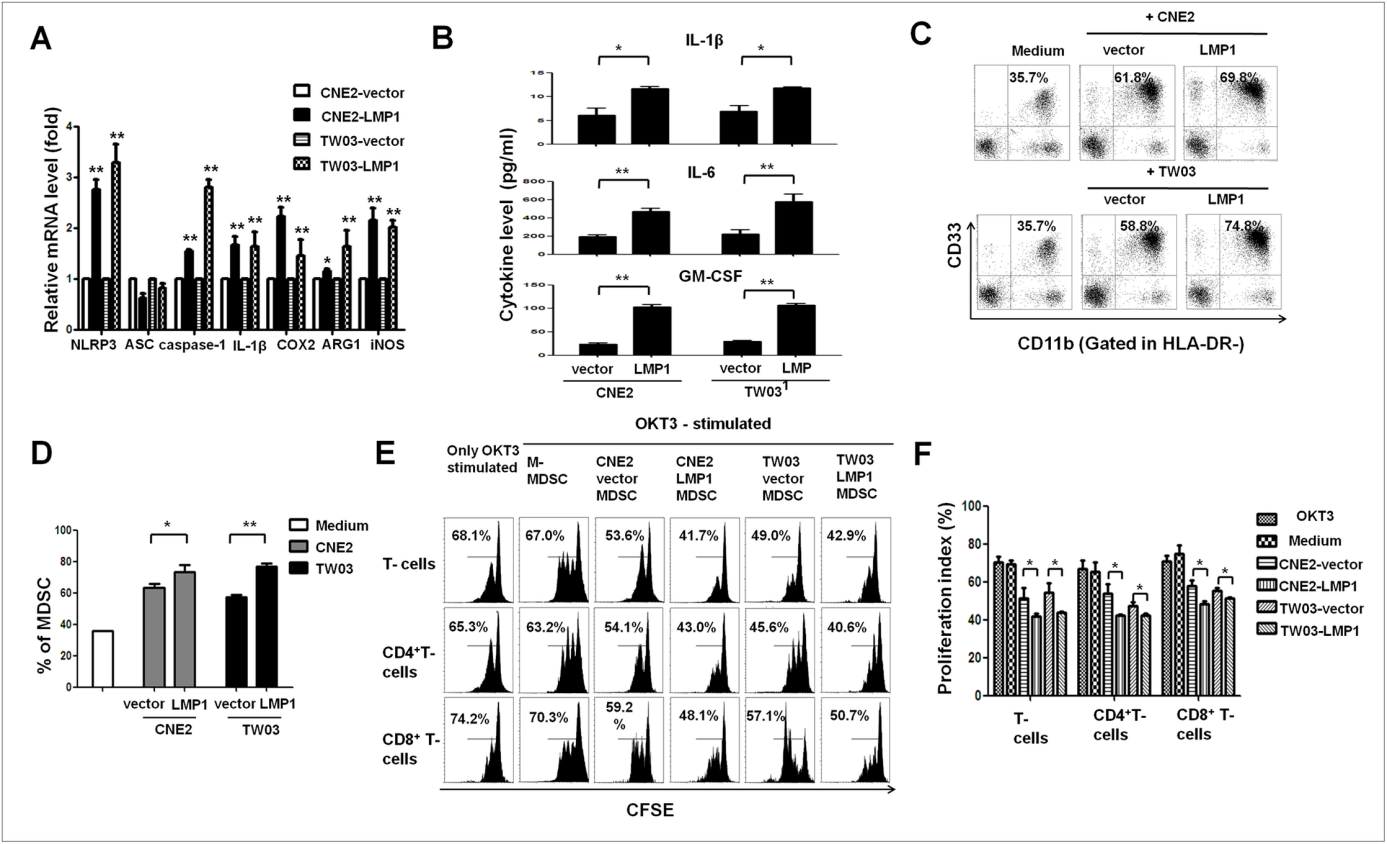
LMP1 promoted NPC-induced MDSC differentiation and the expression of MDSC-related molecules in NPC cells. CD33^+^ cells were isolated from healthy PBMCs using human CD33 MicroBeads and were co-cultured with NPC or NPC-LMP1 cells in a Transwell system for 48 h. The percentage of CD33^+^CD11b^+^HLA-DR^-^ MDSCs was measured by FACS staining. (**A**) Expression of the NLRP3, ASC, caspase-1, IL-1β, COX-2, Arg-1 and iNOS mRNAs was determined via real-time qRT-PCR, and the results were normalized against GAPDH expression. (**B**) NPC cells, including CNE2-vector, CNE2-LMP1, TW03-vector, and TW03-LMP1 cells, were cultured overnight following stimulation with LPS (0.1 μg/mL) and ATP (5 mM, 30 min) or no stimulation. Then, the culture supernatants were collected, and the concentrations of IL-1β, IL-6 and GM-CSF were measured by ELISA. (**C**) Representative density plots are shown as the CD33^+^CD11b^+^cells in the HLA-DR^-^ gate induced by NPC or NPC-LMP1 cells in different combinations. Representative data from 1 of 5 experiments are presented. (**D**) Statistical analysis of the percentage of MDSCs mediated by NPC cells in different combinations. CFSE-labeled PBMCs were co-cultured with M-MDSCs, CNE2-vector-MDSCs, CNE2-LMP1-MDSCs, TW03-vector-MDSCs or TW03-LMP1-MDSCs at a ratio of 1:1 in OKT3-coated 96-well plates. After 3 days, the cells were collected, stained with anti-human mAbs against CD4 and CD8 and quantified by flow cytometry. Representative FACS density plots (**E**) and the statistical data (**F**) showed that the proliferation of PBMCs and CD4^+^ and CD8^+^ T cells was markedly suppressed by CNE2-LMP1-MDSCs or TW03-LMP1-MDSCs compared with the effects of CNE2-vector-MDSCs or TW03-vector-MDSCs. Data are presented as the means ± SEM of representative experiments performed in triplicate. *P < 0.05, **P < 0.01 compared with the control treatment.

In previous reports, the expression of the LMP1 protein in tumor cells has been associated with immune tolerance in EBV-associated cancers [24]. To investigate the role of EBV-LMP1 in the differentiation of tumor-associated MDSCs in NPC, we first measured the mRNA expression of genes related to MDSC differentiation as described in previous reports [25, 26]. NLRP3 inflammasome molecules (NLRP3, apoptosis-associated speck-like protein containing a caspase activation and recruitment (CARD) domain [ASC], caspase-1 and IL-1β), COX-2, arginase 1 (Arg-1) and inducible nitric oxide synthase (iNOS), which are related to MDSC differentiation, were investigated in LMP1-positive and LMP1-negative CNE2 and TW03 cells. We observed increased mRNA expression for NLRP3, caspase-1, IL-1β, COX-2, Arg-1 and iNOS in CNE2-LMP1 and TW03-LMP1 cells compared to expression in control cells (Fig 3A). Moreover, the secretion of cytokines related to MDSC differentiation, including IL-1β, IL-6 and GM-CSF, was increased in CNE2-LMP1 and TW03-LMP1 cells relative to that of CNE2-vector and TW03-vector cells (Fig 3B). In addition, EBV-LMP1 enhanced the generation of functional MDSCs (HLA-DR^-^CD11b^+^CD33^+^ cells) from TW03 and CNE2 cells (Fig 3C–3F and S1 Fig) in a Transwell co-culture system *in vitro* [18].

### GLUT1-dependent glycolysis is essential for LMP1-mediated MDSC induction

**Fig 4:**
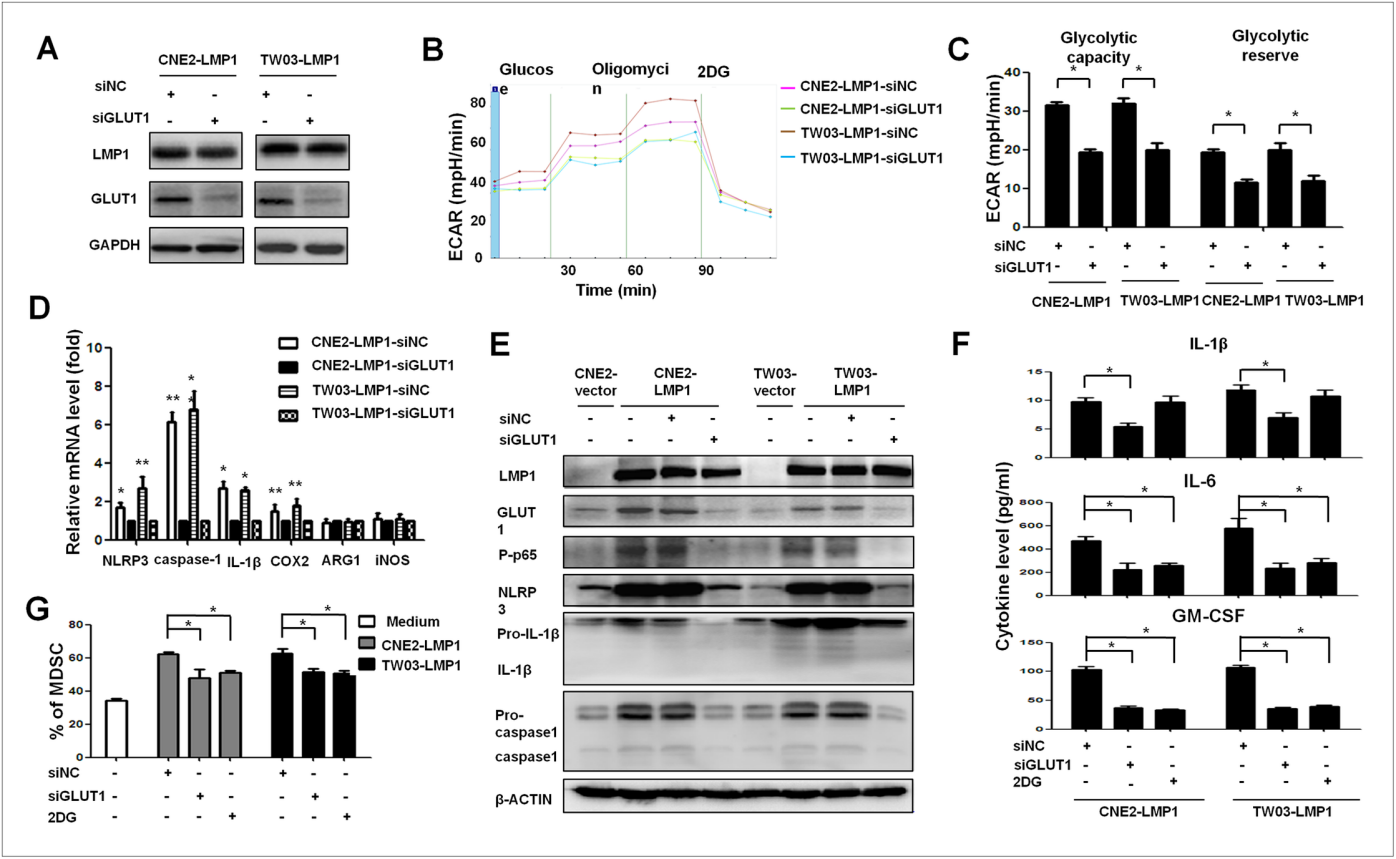
GLUT1-dependent glycolysis was required for NPC-LMP1-mediated MDSC differentiation. (**A**) WB showed the level of GLUT1 in CNE2-LMP1-siNC, CNE2-LMP1-siGLUT1, TW03-LMP1-siNC and TW03-LMP1-siGLUT1 cells, and GAPD was included as the control. (**B** and **C**) Representative ECAR assays for CNE2-LMP1-siNC, CNE2-LMP1-siGLUT1, TW03-LMP1-siControl and TW03-LMP1-siGLUT1 cells. The time course and calculations for (**B**) glycolytic capacity and (**C**) statistical analysis of glycolytic capacity and glycolytic reserve are shown. (**D**) Expression of the NLRP3, ASC, caspase-1, IL-1β, COX-2, Arg-1 and iNOS mRNAs was determined via qRT-PCR in CNE2-LMP1-siControl, CNE2-LMP1-siGLUT1, TW03-LMP1-siControl and TW03-LMP1-siGLUT1 cells. (**E**) Representative immunoblotting of NPC-vector, NPC-LMP1, NPC-LMP1-siNC and NPC-LMP1-siGLUT1 cells stained with the indicated antibodies (n = 5). (**F**) Secretion of the cytokines IL-1β, IL-6 and GM-CSF from NPC-LMP1 cells following treatment with siGLUT1, siControl (siNC) or 2-DG was determined via ELISA. (**G**) Statistical analysis of the percentage of CD33^+^CD11b^+^HLA-DR^-^MDSCs mediated by CNE2-LMP1 or TW03-LMP1 cells following treatment with siGULT1, siNC or 2-DG. Data are presented as the means ± SEM of representative experiments performed in triplicate. *P < 0.05, **P < 0.01 compared with the control treatment.

As mentioned in the Introduction, the metabolic reprogramming of malignant cells, which results in the enhancement of extra-mitochondrial glycolysis, can be sufficient for the induction of neighboring MDSCs [14]. To investigate whether the impact of LMP1 on MDSCs was dependent on enhanced extra-mitochondrial glycolysis, we blocked GLUT1, which is upstream of all glycolytic pathways. The GLUT1 protein belongs to the solute carrier 2A (SLC2A) family of glucose transporter molecules and is a key regulator of glycolysis [27]. Here, the glucose consumption and lactate production rates were substantially decreased when endogenous GLUT1 expression was knocked down with siRNA targeting GLUT1 in CNE2-LMP1 and TW03-LMP1 cells, as shown by ECAR measurements, and the effects included changes in the glycolytic capacity and glycolytic reserve (Fig 4A–4C). In addition, GLUT1 knockdown decreased the expression of glycolysis-related genes, including HK2, GPI, and PFKFB2 and 3, in CNE2-LMP1 and TW03-LMP1 cells (S2A Fig). Interestingly, the mRNA levels of MDSC differentiation-related genes, including NLRP3, caspase-1, IL-1β, and COX-2, were decreased in CNE2-LMP1 and TW03-LMP1 cells after blockade of endogenous GLUT1 expression (P < 0.05, Fig 4D). In addition, the expression of the P-p65, NLRP3, pro-caspase-1 and pro-IL-β proteins and the self-cleavage of caspase-1 and IL-1β were increased in CNE2-LMP1 and TW03-LMP1 cells compared with control cells; however, the increased expression of P-p65, NLRP3, pro-caspase-1 and pro-IL-β and the self-cleavage of caspase-1 and IL-1β were reversed after GLUT1 blockade in CNE2-LMP1 and TW03-LMP1 cells (Fig 4E). Subsequently, the secretion of the cytokines IL-1β, IL-6 and GM-CSF was significantly decreased in CNE2-LMP1 and TW03-LMP1 cells following treatment with siGLUT1, and IL-6 and GM-CSF levels were significantly decreased after treatment with 2-DG (Fig 4F).

Importantly, the level of HLA-DR^-^CD11b^+^CD33^+^ MDSC induction mediated by CNE2-LMP1 and TW03-LMP1 cells was sharply decreased following treatment with siGLUT1 and 2-DG (Fig 4G, P < 0.05). Meanwhile, the induction of CNE2-derived HLA-DR^-^CD11b^+^CD33^+^ MDSC differentiation was increased in low pH medium (S2B Fig). The expression level of suppressive molecules, including Arg-1, iNOS, programmed death-ligand 1 (PD-L1) and phosphorylated signal transducer and activator of transcription 3 (P-STAT3), were decreased in HLA-DR^-^CD11b^+^CD33^+^ MDSCs induced by CNE2-LMP1-siGLUT1 or TW03-LMP1-siGLUT1 cells instead of CNE2-LMP1 or TW03-LMP1. Moreover, the expression levels of these suppressive molecules, including Arg-1, iNOS, PD-L1 and P-STAT3, were restored during CNE2-mediated MDSC differentiation in low pH medium (S2C Fig). These data indicate that GLUT1-dependent glycolysis is involved in NPC-induced MDSC differentiation. We altered glucose metabolism using metformin [28, 29] to further confirm the relationship between GLUT1-dependent glycolysis and LMP1-mediated MDSC differentiation and discovered that LMP1-mediated MDSC differentiation and GLUT1 expression were attenuated following the administration of metformin, as shown in S2D and S2E Fig. Taken together, these observations indicate that GLUT1-dependent glycolysis promotes NPC-derived MDSC differentiation, which is a process associated with the activation of the NLRP3 inflammasome.

### NLRP3 inflammasome activation contributes to LMP1-mediated MDSC expansion

**Fig 5:**
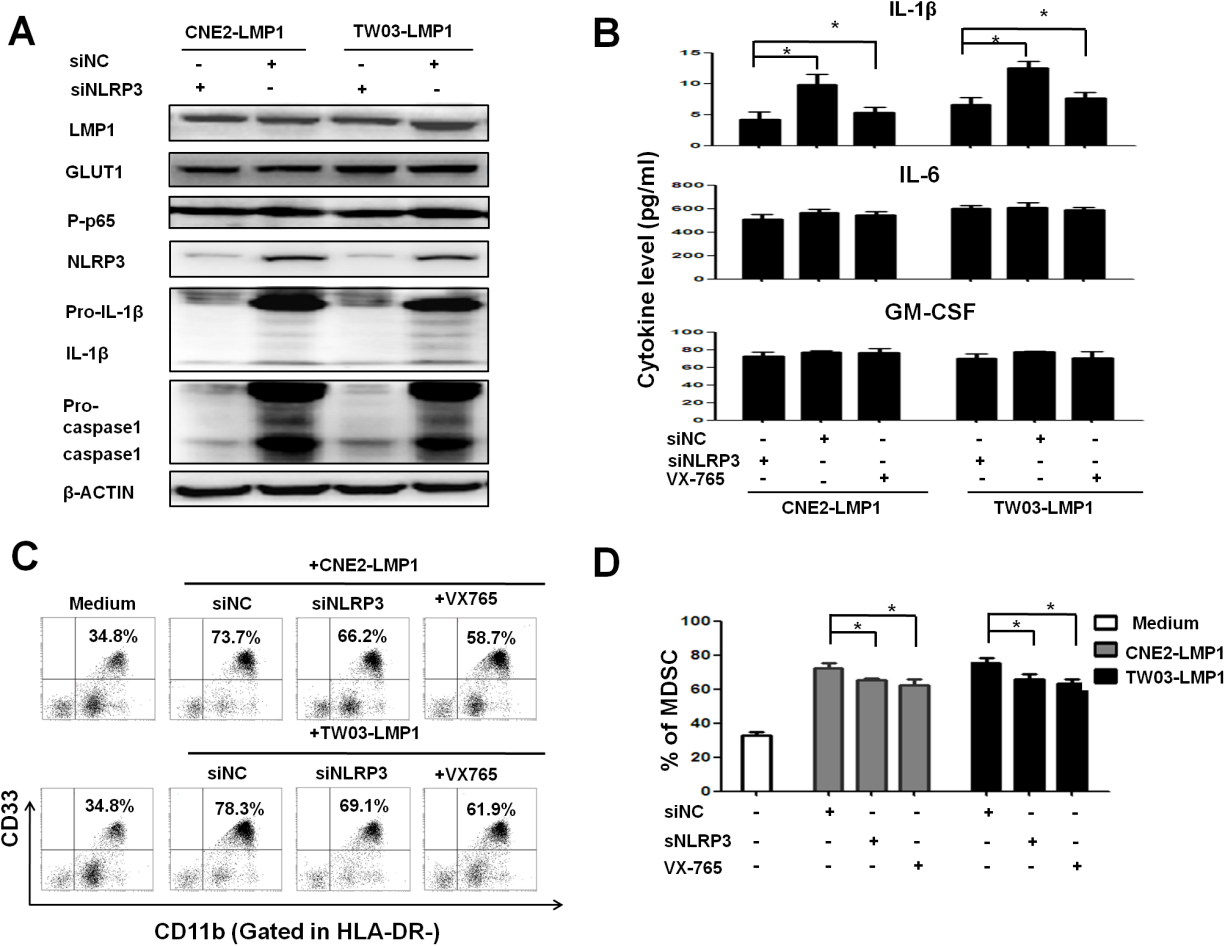
NLRP3 expression was involved in the accumulation of NPC-LMP1-mediated MDSCs. (**A**) Representative immunoblots of CNE2-LMP1 and TW03-LMP1 cells treated with siNC or siNLRP3 and stained with the indicated antibodies (n = 3). (**B**) Secretion of the cytokines IL-1β, IL-6 and GM-CSF from NPC-LMP1 cells following treatment with si-NLRP3, siControl (siNC) or VX-765 was determined via ELISA. (**C**) Blockade of NLRP3 decreased the differentiation of HLA-DR^-^CD11b^+^CD33^+^ MDSCs mediated by NPC-LMP1. Representative data from 5 independent experiments are shown. (**D**) Statistical analysis of the percentage of CD33^+^CD11b^+^HLA-DR^-^MDSCs mediated by CNE2-LMP1 or TW03-LMP1 cells following treatment with si-NLRP3, siNC or VX-765. Representative data from 3 independent experiments are shown.

Because GLUT1-dependent glycolysis activated the phosphorylation of p65 (NF-κB) and the NLRP3 inflammasome, we knocked down NLRP3 in CNE2-LMP1 and TW03-LMP1 cells using siRNA specific for NLRP3. The expression of EBV-LMP1, GLUT1 and P-p65 in CNE2-LMP1 and TW03-LMP1 cells was not affected by the siNLRP3 treatment, whereas the levels of pro-IL-1β and pro-caspase-1 were dramatically decreased once endogenous NLRP3 expression was blocked, without any effect on the self-cleavage of caspase-1 and IL-1β (Fig 5A). In addition, NLRP3 knockdown and the administration of the small molecule VX765 (caspase-1 inhibitor) significantly decreased the secretion of IL-1β but not of IL-6 and GM-CSF (Fig 5B). Importantly, our data show that blocking NLRP3 or inhibiting caspase-1 both substantially attenuate LMP1-promoted MDSC differentiation (Fig 5C and 5D). These data indicate that activation of the NLRP3 inflammasome is involved in the generation of LMP1-derived MDSCs and is dependent on IL-1β production.

### LMP1 interacts with GLUT1 to disrupt GLUT1 p62-dependent autolysosomal degradation

**Fig 6:**
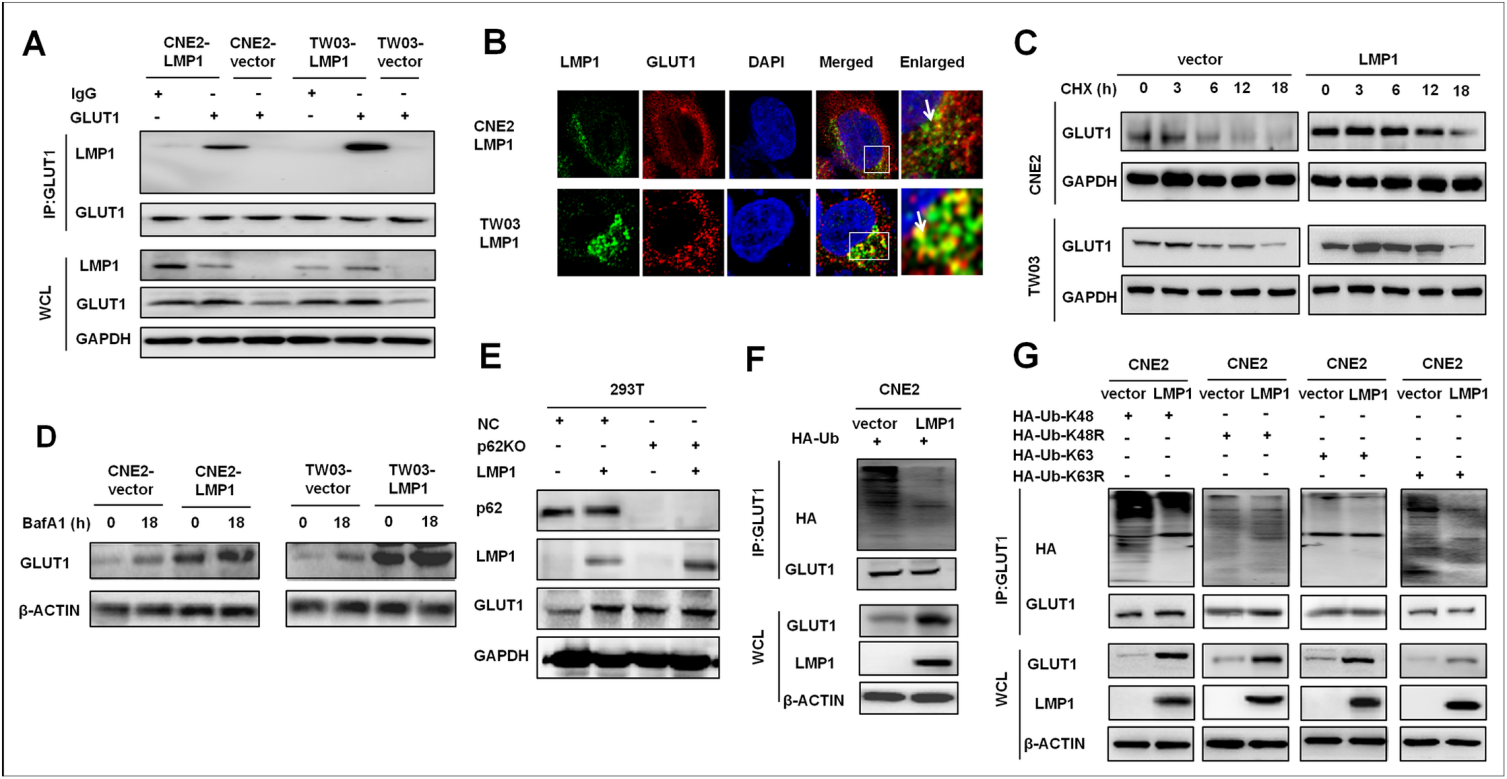
LMP1 co-expression with GLUT1 in NPC-LMP1 cells stabilized GLUT1 proteins by disrupting GLUT1 K48 ubiquitination and p62-dependent lysosomal degradation. (**A**) LMP1 physically interacts with endogenous GLUT1. NPC-LMP1 and NPC-vector cells were treated with MG-132 (1 μM) for 4 h to stabilize GLUT1, followed by immunoprecipitation (IP) with an anti-GLUT1 antibody. Immunoblotting was performed using anti-LMP1 and anti-GLUT1 antibodies. Anti-IgG was used as a negative control for IP. Representative data from 5 independent experiments are shown. (**B**) Immunofluorescence detection of EBV-LMP1 and GLUT1 in stable NPC-LMP1 cell lines. NPC-LMP1 and NPC-vector cells were seeded onto a chambered cover glass overnight. LMP1 and GLUT1 were detected with LMP1 and GLUT1 antibodies, nuclei were stained with DAPI, and confocal images were analyzed using ImageJ software. (**C**) NPC-LMP1 and NPC-vector cell lines were treated with CHX for 18 h. Proteins were harvest at 0, 3, 6, 12 and 18 h, and the expression of GLUT1 was measured via immunoblotting. Representative data from 5 independent experiments are shown, and GAPDH was included as a control. (**D**) NPC-LMP1 and NPC-vector cell lines were treated with the lysosome inhibitor BafA1 for 12 h, proteins were harvested at 0 and 12 h, and GLUT1 expression was measured by immunoblotting. Representative data from 5 independent experiments are shown, and GAPDH was included as a control. (**E**) Immunoblotting showed that GLUT1 was expressed at higher levels in 293T cells than in p62KO-293T cells, whereas GLUT1 was expressed at higher levels in 293T-LMP1 cells than in 293T cells but was not found at higher levels in p62KO-293T-LMP1 cells compared with p62KO-293T cells. Representative data from 1 of 5 experiments are shown. (**F-G**). NPC-LMP1 and NPC-vector cell lines cultured in 6-well plates were transfected with hemagglutinin (HA)-tagged Ub (4 μg/well) (**F**) HA-tagged Ub-K48, HA-tagged Ub-K48R, HA-tagged Ub-K63 or HA-tagged Ub-K63R (**G**) and then treated with 20 mM MG132 for 6 h prior to harvest. Cell lysates were immunoprecipitated with anti-HA antibodies and then subjected to WB with an anti-GLUT1 antibody to measure the levels of ubiquitinated GLUT1 proteins (upper panels). WCLs were blotted to evaluate the levels of GLUT1 proteins (lower panels). β-actin expression was used as a protein loading control. The experiment shown is a representative of three independent experiments.

We observed that EBV-LMP1 colocalized and interacted with GLUT1 in the cytoplasm and membranes of NPC-LMP1 cells using co-immunoprecipitation (co-IP) and fluorescence immunostaining assays *in vitro* (Fig 6A and 6B). Importantly, we discovered that the half-life of GLUT1 was longer in CNE2-LMP1 and TW03-LMP1 cells than in control cells in the cycloheximide (CHX) ‘‘chase” assays (Fig 6C). This mechanism contributed to the higher cellular concentration of GLUT1 and the up-regulation of GLUT1 mRNA expression, as shown in Fig 2D. To date, at least three systems of protein degradation, including the proteasome, lysosome, and autolysosome pathways, have been described [30]. According to the results from the mechanistic analysis, the proteasome inhibitor MG132 increased the half-life of GLUT1 in both CNE2-LMP1 and TW03-LMP1 cells and in CNE2-vector and TW03-vector cells, whereas the autophagy inhibitor bafilomycin A1 (BafA1) increased the half-life of GLUT1in CNE2-vector and TW03-vector cells but not in CNE2-LMP1 and TW03-LMP1 cells (Fig 6D and S3A and S3B Fig). We further discovered that GLUT1 levels were higher in p62KD-293T cells than in 293T cells, whereas GLUT1 levels were increased in 293T cells with forced LMP1 expression but not in p62KD-293T cells with forced LMP1 expression (Fig 6E). Moreover, approximately the same amount of GLUT1 protein was pulled down by Flag-p62 in the co-IP analysis as in the CNE2-vector and CNE2-LMP1 cells transfected with Flag-p62 plasmids, although GLUT1 levels were increased in CNE2-LMP1 cells compared with CNE2-vector cells, as determined by an immunoblotting analysis (S3C Fig). These data suggest that LMP1 inhibits GLUT1 autophagic degradation in a p62-dependent manner by disrupting the interaction of GLUT1 with the autophagic cargo receptor p62. In addition, a dramatic decrease in ubiquitinated GLUT1 was observed in CNE2-LMP1 cells compared to that in CNE2-vector cells (Fig 6F). Meanwhile, the expression levels of GLUT1 in CNE2-LMP1 cells were much higher than the levels in CNE2 cells, suggesting that LMP1 might affect GLUT1 K48-linked ubiquitination. To confirm this hypothesis, we employed ubiquitin mutant plasmids (as described in the Methods section) pHA-Ub-K48R (only K48 affected), pHA-Ub-K63R (only K63 affected), pHA-Ub-K48 (only K48 retained) or pHA-Ub-K63 (only K63 retained). Transfection with pHA-Ub-K48R resulted in the same level of ubiquitinated GLUT1 in the CNE2-LMP1 and CNE2-vector cell lines, whereas transfection with pHA-Ub-K48 resulted in a lower level of ubiquitinated GLUT1 in CNE2-LMP1 cells than in CNE2-vector cells. Transfection with pHA-Ub-K63R did not result in the expression of ubiquitinated GLUT1 in CNE2-LMP1 and CNE2-vector cells, while transfection with pHA-Ub-K63 resulted in a reduction in ubiquitinated GLUT1 in CNE2-LMP1 cells compared to that in CNE2-vector cells (Fig 6G). Overall, these data suggest that LMP1 disrupts the K48-linked ubiquitination of GLUT1 and the binding of GLUT1 and the autophagic cargo receptor p62 to delay the autolysosomal degradation of the GLUT1 protein.

## Discussion

The differentiation and expansion of MDSCs has been described in various cancers, including NPC [18, 31, 32]. Several oncogenic viruses, including HIV and HCV, have been associated with MDSC expansion in virus-associated cancers [33, 34]. However, researchers have not determined whether viral genes are involved in MDSC differentiation to induce anti-tumor immune suppression in EBV-associated NPC, and its molecular mechanism is largely unknown. For the first time, we have shown a triple positive correlation between the expression of LMP1 and GLUT1 in malignant cells and the abundance of CD33^+^ MDSCs in the stroma of NPC tumors. We showed that LMP1 reprograms GLUT1-dependent extra-mitochondrial glycolysis in NPC cells *in vitro*. This metabolic reprogramming resulted in increased expression of the NLRP3 inflammasome, COX-2 and P-p65 and a subsequent increase in IL-1β, IL-6 and GM-CSF production. Finally, these changes in the environment of malignant cells results in enhanced MDSC induction. One key step is the physical interaction of LMP1 with GLUT1, which blocks its K48-ubiquitination and p62-dependent autolysosomal degradation.

One merit of our report is that we provide an integrated view of the relationship between the expression of LMP1, metabolic reprogramming and the expansion of intra-tumor MDSCs. Previous publications, including one of our reports, have pointed out the pejorative prognostic value of abundant LMP1 and GLUT1 expression, as well as the abundant infiltration of CD33^+^ MDSCs [18]. LMP1 is associated with poorer survival in EBV-associated cancers, including NPC, EBV^+^ B-cell lymphoma and NK/T lymphoma [35–37]. Similarly, the expression of GLUT1 is correlated with shorter survival times in different types of solid tumors and in Hodgkin’s lymphoma [38–41]. Recent studies have shown that in malignant NPC cells, EBV-encoded LMP1 increases extra-mitochondrial glycolysis through the up-regulation of HK2 or the repression of the HoxC8 gene. These metabolic changes were shown to promote tumor progression and resistance to radiotherapy [10–12, 42]. Our results are consistent with these previous findings but also go beyond them by showing the correlation between LMP1/GLUT1 expression and CD33^+^ MDSC infiltration, their negative prognostic value and their causal relationship.

Notably, the induction of glycolysis by LMP1 was attenuated following the blockade of endogenous GLUT1 expression or the inhibition of glucose consumption by 2-DG. Many studies have linked the abundance of GLUT proteins in the cell membrane and the activity of intracellular enzymes such as HKs, which results in the concomitant intracellular accumulation of glucose and alterations in glycolysis [43–45]. We consistently observed a decrease in the expression of various glycolytic genes, including HK2 and PFKB2 and 3, in GLUT1-knockdown CNE2-LMP1 and TW03-LMP1 cells (S2C Fig). Interestingly, we discovered that LMP1 not only induces GLUT1 mRNA expression but also increases the protein level of GLUT1 by interacting with GLUT1 to disrupt GLUT1 K48-ubiquitination and p62-dependent autolysosomal degradation, which results in GLUT1 protein stabilization and glucose import to increase the rate of glycolysis. This result is consistent with the findings of another study of B-cell lymphoma that showed that LMP1 promoted GLUT1 plasma membrane localization via IKKβ expression and activation of the NF-κΒ signaling pathway, which promoted EBV-infected B-cell lymphoma proliferation and apoptosis resistance by driving glucose import and increasing glycolysis [46]. In addition, LMP1 has been shown to promote GLUT1 mRNA and protein expression in NPC cells through activation of the p65 subunit of the NF-κB pathway. In this study, the C-terminal activating region 2 (CTAR2) of LMP1 appears to be the key domain involved in activating mammalian target of rapamycin complex 1 (mTORC1) and NF-κB, mainly through inhibitor of κB kinase (IKK)-mediated phosphorylation of tuberous sclerosis complex 2 (TSC2) at Ser93 [47]. This finding is consistent with our observations that pharmacological NF-κB inhibition in NPC-LMP1 cells decreased the level of P-p65 and the GLUT1 mRNA and protein levels. Simultaneously, this treatment induced a decrease in IL-1β and IL-6 secretion and NPC-induced MDSC differentiation (S4 Fig). Moreover, LMP1 promoted an increase in GLUT1 protein levels by directly binding to GLUT1 through amino acids (aas) 230–322 of LMP1, which is located between CTAR1 and CTAR2 (maybe in CTAR3 aas275-330), thus leading to the inhibition of K48-ubiquitination of GLUT1 and p62-dependent lysosomal degradation (S5 Fig).

The consequence of these multiple modifications is a higher rate of glycolysis in LMP1-positive than in LMP1-negative cells. The contribution of LMP1 to modulating immune responses in EBV^+^ tumors remains controversial. Some studies suggest that the immune evasion signals mediated via LMP1 may shield an EBV-infected cell from host immune monitoring in the latent infection phase [48]. In contrast, LMP1 is suspected to enhance antigen processing and presentation through the up-regulation of TAP and MHC class I in Burkitt lymphoma cells. This is probably very important for the host-virus relationship of healthy EBV carriers in which the elimination of EBV-infected B cells expressing the transforming latency III (growth) program is crucial. LMP1 expression in these cells might facilitate their immune recognition and elimination, which is essential for the survival of the host [49–51]. In contrast, we provide one the first demonstrations of the immunosuppressive action of LMP1 in an EBV-related malignancy with a detailed account of the underlying molecular and cellular steps. MDSCs are the precursors of immune cells of myeloid origin, including macrophages, dendritic cells and neutrophils, and are correlated with the suppression of anti-tumor immunity [52]. Their differentiation and the stabilization of their phenotype requires the action of a series of cytokines, including IL-1β, IL-6 and GM-CSF. We show that the induction of these cytokines by LMP1 involves COX-2, Arg-1, iNOS and the NLRP3 inflammasome (NLRP3, caspase-1 and IL-1β). LMP1 expression promotes the phosphorylation of p65 and the activation of the NLRP3 inflammasome, resulting in IL-1β, IL-6 and GM-CSF production (S6 Fig). Strikingly, the production of these cytokines is decreased by the blockade of GLUT1 or the administration of 2-DG, followed by a decrease in glycolysis in NPC cells. To our knowledge, previous reports on the link between glycolysis and the differentiation of tumor-infiltrating MDSCs were not as clear. One study showed that glycolytic activation through the mTOR-hypoxia-inducible factor-1α (HIF-1α) pathway promoted the late differentiation of MDSCs into M1 type macrophages [14], whereas other studies confirmed that enhancing glycolysis is required for MDSC differentiation from precursor cells [53, 54]. Here, our *in vitro* data show that GLUT1-dependent glycolysis, which was promoted by LMP1, is required for LMP1-mediated tumor-associated MDSC differentiation. The attenuation of glycolysis by siLGUT1, 2-DG or the pharmaceutical drug metformin decreased the extent of NPC-LMP1-induced MDSC differentiation (S2D Fig), whereas low pH medium increased CNE2-derived MDSC differentiation *in vitro* (S2B Fig). These findings might inspire novel strategies for treating patients with NPC. In particular, metformin could be used to prevent and treat cancer, based on its different mechanisms [55].

The inflammasome is a protein complex comprising an intracellular sensor (typically a Nod-like receptor such as NLRP3), the precursor pro-caspase-1 and the adaptor ASC [56]. Physiologically, NLRP3 is activated by various danger signals, including those produced by bacterial species such as *Salmonella*, which results in the maturation of caspase-1 and the processing of its substrates, IL-1β and IL-18 [56]. Disruption of glycolysis and decreased ATP production have been reported to activate the NLRP3 inflammasome, and the activation of NLRP3 has an important effect on the differentiation and function of innate immune cells, such as macrophages [57, 58]. Recently, one report found that PKM2-dependent glycolysis promotes NLRP3 and AIM inflammasome activation in macrophages [59]. In the present study, our results confirm that LMP1-GLUT1-dependent glycolysis triggers the activation of the NLRP3 inflammasome, which was associated with the production of IL-1β from NPC cells. The suppression of NLRP3 inflammasome activity by siNLRP3 or the caspase-1 inhibitor VX765 significantly attenuated LMP1-mediated MDSC induction *in vitro*. Other groups have found that in NLRP3^-/-^ mice, the number of tumor-associated MDSCs was significantly decreased, and the mice displayed a better response to dendritic cell vaccination against melanoma than wild-type mice [60]. Furthermore, the chemotherapeutic agents gemcitabine (Gem) and 5-fluorouracil (5FU) have been confirmed to activate the NLRP3 inflammasome in MDSCs to curtail anticancer immunity, and Gem and 5FU exert greater anti-tumor effects when tumors are established in NLRP3^-/-^ or casp1^-/-^ mice [61]. Overall, these data indicate that the activation of the NLRP3 inflammasome has an important role in functional tumor-associated MDSC induction. However, several studies have shown that EBV infection can induce the NLRP3 inflammasome to produce IL-1β and IL-18, leading to the growth and activation of anti-apoptosis mechanisms of EBV-infected tumor cells [62–64]. In the present study, we showed for the first time that LMP1 can induce NLRP3 expression and the activation of the NLRP3 inflammasome in NPC cells through GLUT1-dependent glycolysis. In addition, our data reveal that LMP1 up-regulates COX-2 expression and the NF-κB pathway (P-p65), which induced IL-6 and GM-CSF secretion, as previously reported by our group and other researchers [18, 65, 66]. Therefore, LMP1-mediated MDSC expansion is dependent on GLUT1-dependent glycolysis to activate multiple signaling pathways.

**Fig 7:**
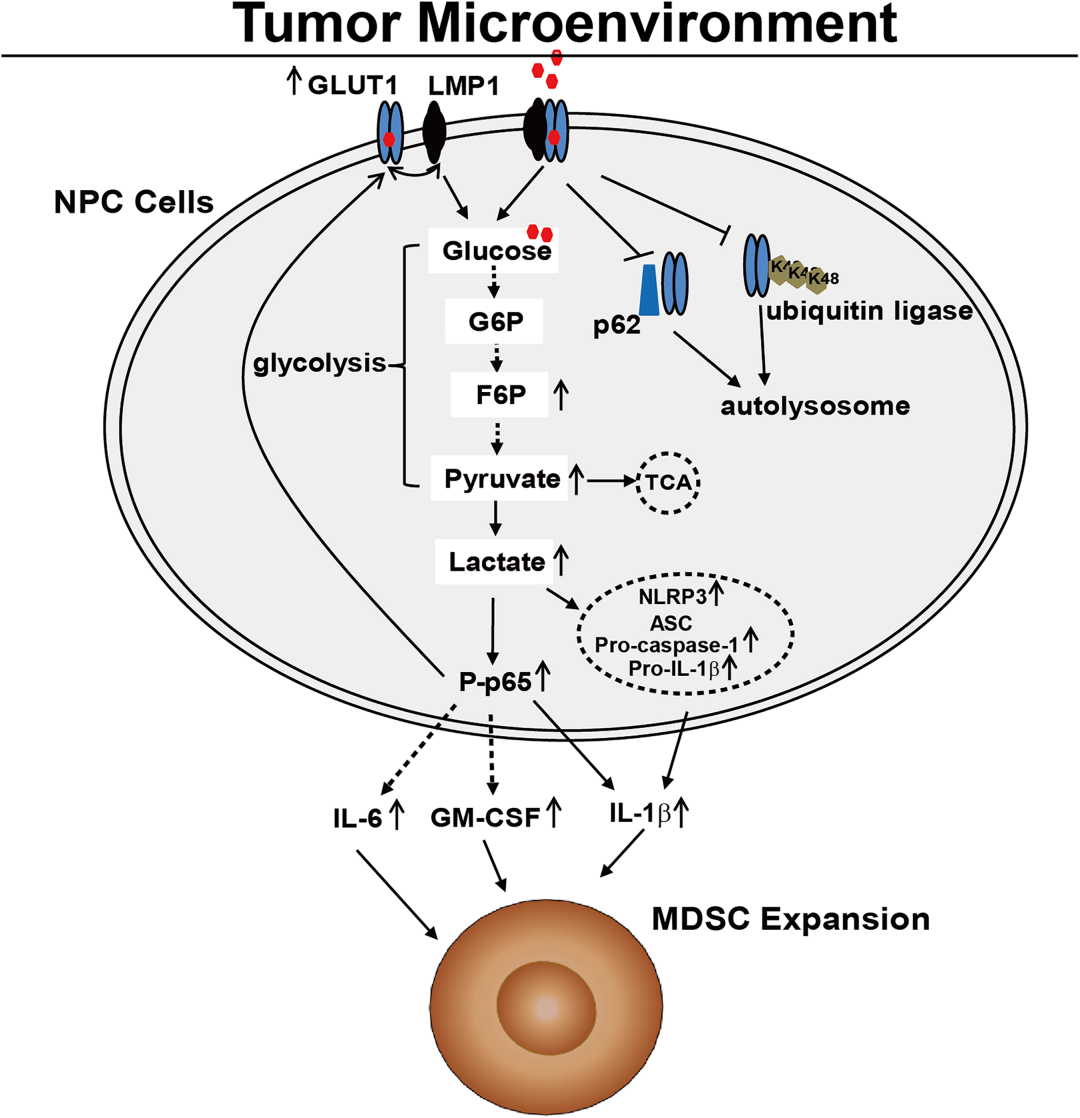
A schematic diagram illustrating the molecular signaling pathways regulating LMP1-mediated MDSC differentiation in NPC. LMP1 up-regulates GLUT1 expression by activating p65 and binding to GLUT1 to delay GLUT1 p62-dependent autolysosomal degradation. The increased GLUT1 expression results in more extensive glycolysis. The increase in glycolysis triggers the activation of p65 and the NLRP3 inflammasome signaling pathway, leading to the production of IL-1β, IL6 and GM-CSF from NPC cells, which promotes tumor-associated MDSC expansion in NPC microenvironments.

In conclusion, our results provide new insights into the molecular mechanism of tumor-induced MDSC expansion and immune evasion in EBV-associated NPC. In terms of clinical and pathological observations, we report a positive correlation between LMP1/GLUT1 expression in malignant cells and the abundance of CD33^+^ MDSCs in the NPC tumor microenvironment. In terms of molecular and cellular mechanisms, we show that LMP1 interacts with GLUT1 to delay GLUT1 p62-dependent autolysosomal degradation and increase GLUT1-dependent glycolysis *in vitro*. Moreover this effect on p62-dependent autolysosomal degradation contributes to LMP1-mediated MDSC induction (S7 Fig). Increased glycolysis promotes tumor-associated MDSC expansion in the NPC microenvironment by triggering the activation of p65 and the NLRP3 inflammasome signaling pathway, which leads to the production of IL-1β, IL6 and GM-CSF by malignant NPC cells (Fig 7). These findings indicate an important role for the EBV antigen LMP1 in immuno-metabolism and immune evasion and will guide the future development of therapeutic strategies for treating NPC.

## Materials and methods

### Ethics statement

Paraffin-embedded tumor tissues were collected from 112 patients with newly diagnosed NPC at the Sun Yat-sen University Cancer Center, Guangzhou, China from March 2011 to July 2012. The patients’ clinical information is provided in S1 Table. Written informed consent was provided by all patients before the tumor biopsies were obtained. This study was conducted in accordance with the Declaration of Helsinki and approved by the Research Ethics Committee of the Sun Yat-sen University Cancer Center.

TW03 and CNE2 are EBV-negative NPC cell lines, and TW03, CNE2, human embryonic kidney cell line 293T and p62KO-239T cells were all maintained in complete RPMI 1640 medium supplied with 10% fetal bovine serum (FBS, Life Technologies).

### Antibodies and reagents

Detailed information about the antibodies and reagents used in this study is shown in S3 Table.

### Plasmids, lentiviruses and transduction

The pcDNA3.1-LMP1 expression plasmid was constructed by cloning the entire EBV-LMP1 coding sequence into the pcDNA3.1 vector for transient expression. The EBV-LMP1 coding sequence was also inserted into a lentivirus vector, pLJM1-EGFP (GeneCopoeia), and the lentiviral expression construct and the packaging plasmid mix were cotransfected into 293T cells to generate recombinant lentiviruses according to the manufacturer’s instructions. For the generation of the stable LMP1-expressing NPC cell lines CNE2-LMP1 and TW03-LMP1, the NPC cell lines were infected with recombinant lentivirus-transducing units plus 8 μg/mL Polybrene (Abbott Laboratories Corp.) and incubated for 16 h. At 48 h after infection, the medium was replaced with fresh medium containing 1 μg/mL puromycin and cultured for another 6 days to establish the NPC cell lines stably expressing LMP1.

### siRNA transfection

To alter GLUT1 and NLRP3 levels in CNE2-LMP1 and TW03-LMP1 cells, the cell lines were cultured to 50–60% confluence and were then transfected with GLUT1-siRNAs or NLRP3-siRNAs. Chemically synthesized 21-nt siRNA duplexes were obtained from GenePharma Bio and transiently transfected into NPC cells using Lipofectamine 2000 (Invitrogen) according to the manufacturer’s instructions. A siRNA targeting a control gene (siNC) was included in this study. After transfection for 24 or 48 h, the mRNA levels in the cells were measured via RT-PCR, and specific protein expression was detected via Western blotting (WB). The sequences of siGLUT1 and NLRP3 are as follows: siGLUT1_001 5ˈ-CCAAGAGTGTGCTAAAGAA-3ˈ; siGLUT1_002 5ˈ- GCATGTGCTTCCAGTATGT-3ˈ; siGLUT1_003 5ˈ- AGAAGGTGATCGAGGAGTT-3ˈ; siNLRP3_001, 5ˈ- GAAATGGATTGAAGTGAAA-3ˈ; siNLRP3_002, 5ˈ- GGATCAAACTACTCTGTGA-3ˈ; and siNLRP3_003, 5ˈ- GGAGAGACCTTTATGAGAA-3ˈ.

### Quantitative real-time RT-PCR (qRT-PCR)

RNA was extracted from cells using TRIzol reagent (Invitrogen) according to the manufacturer’s protocol and used for cDNA synthesis (Prime Script RT Master Mix, TaKaRa Bio). All qRT-PCR products were amplified using Power SYBR Green (Life Technologies) and specific primers (S4 Table). PCR reactions and data analysis were performed on a Step One Real-Time PCR System (Applied Biosystems) using the comparative Ct method and the housekeeping gene β-actin.

### Tumor-associated MDSC induction *in vitro*

Tumor-associated MDSCs were generated from CD33^+^ cells isolated by anti-CD33 beads (Miltenyi Biotec Company) from peripheral blood mononuclear cells (PBMCs) of healthy donors in a co-culture Transwell system (0.4-μm pore, Corning) with the NPC cell lines TW03, CNE2, TW03-LMP1 or CNE2-LMP1 as previously described. Several parallel wells were then treated with siGLUT1, siNLRP3, 2-DG, the caspase-1 inhibitor VX-765 (Selleck, 50 μM) or metformin (Sigma-Aldrich, 40 mM). HLA-DR^-^CD11b^+^CD33^+^ cells were defined as MDSCs and measured using a fluorescence-activated cell sorting (FACS) analysis. The suppression of MDSC on T cells was analyzed by co-culturing cells with carboxyfluorescein diacetate succinimidyl ester (CFSE, 10 μM, Molecular Probes)-labeled T cells obtained from healthy donors in 96-well plates that had been pre-coated with OKT-3 (1 μg/mL, R&D Systems) at a ratio of 1:1. T cells were harvested after 72 h and stained for the cell surface markers CD3, CD4, and CD8. Data were obtained and analyzed using FACS.

### Cytokine production (ELISA)

Cytokines, including IL-1β, IL-6 and GM-CSF, from NPC cell lines or NPC-LMP1 cell lines were assessed after treatment with siGLUT1, siNLPR3, 2-DG or the caspase-1 inhibitor VX765 for 24 h. Supernatants were collected, and IL-1β, IL-6 and GM-CSF were measured using cytokine ELISA kits (eBioscience) following the manufacturer’s instructions.

### Flow cytometry, immunoprecipitation and WB analysis

For FACS analysis, single-cell suspensions were stained with the appropriate fluorescent antibodies according to the manufacturer’s instructions. Fluorescent antibodies used to stain human cell surface markers were purchased from BD Biosciences or eBioscience. Detailed information about the fluorescent antibodies is shown in S3 Table.

For WB analyses, cell lysates were separated via 8% or 10% SDS-PAGE, transferred to PVDF membranes (Millipore), blocked, and incubated in different primary antibodies, including specific antibodies. Then, the membranes were incubated in horseradish peroxidase (HRP)-conjugated secondary antibodies (Santa Cruz Biotechnology). The protein bands were visualized using an ECL detection kit (Perkin Elmer Life Science). Cycloheximide (CHX, Biosharp, 10 μM), MG132 (Sigma-Aldrich, 10 μM), and BafA1 (Sigma-Aldrich, 10 μM) were used to pre-treat the cells and inhibit new protein synthesis or protein degradation via the proteasome or lysosome systems.

For immunoprecipitation (IP), whole-cell extracts were precleared with protein A/G beads (Pierce) for 30 min and incubated with specific antibodies at 4°C overnight. After 2 h, 25 μL of a 1:1 slurry of Protein A/G Plus agarose beads was added, and samples were incubated for an additional 2 h. The immunoprecipitates were washed five times with a low-salt lysis buffer and eluted with 3X SDS Loading Buffer, followed by SDS-PAGE analysis. Proteins were transferred to polyvinylidene fluoride (PVDF) membranes (Bio-Rad) and further incubated with the appropriate antibodies. The LumiGlo Chemiluminescent Substrate System (KPL) was used for protein detection. Detailed information about the antibodies used for IP and WB is shown in S3 Table.

### Metabolic flux measurements

ECAR, a measurement of lactate production, was determined upon re-addition of glucose using an extracellular flux analyzer (XF96, Seahorse Bioscience), as described in the manufacturer’s instructions. Briefly, cells were seeded in the detection plate at a density of 2.5 to 3X10^4^ cells per well, incubated with XF media (Seahorse Bioscience) lacking glucose for 12 h and monitored under basal conditions with no added glucose or glutamine and in response to 10 mM glucose, 10 μM oligomycin (Seahorse Bioscience), and 100 μM 2-DG (Sigma-Aldrich). ECAR values were normalized to the number of cells. Glycolytic capacity was defined as the difference between ECAR following the injection of oligomycin and the basal ECAR reading. Glycolytic reserve was defined as the difference in ECAR between the glucose and oligomycin injections.

### Immunofluorescence and IHC staining

Cells were cultured on a glass-bottom dish and directly observed. For immunofluorescence staining, cells grown on gelatin-coated coverslips were fixed with 4% paraformaldehyde, permeabilized using 50 μg/mL digitonin, and then stained with specific antibodies as described previously [30]. Fluorescence was observed and photographed under a confocal fluorescence microscope (Leica TCS SP2). IHC staining for GLUT1 and LMP1 in NPC specimens was performed using primary mouse anti-human LMP1 (Gene Tech) and rabbit anti-human GLUT1 (Novus Biologicals) antibodies according to the manufacturer’s instructions. The slides were scored independently by two pathologists. The samples were scored with IHC values of 0, 1, 2, or 3 when the following percentages of cells were LMP1 or GLUT1 positive: < 5% (0); ≥ 5% but < 10% (1); ≥ 10% but < 50% (2); or ≥ 50% (3). Mouse IgG1 (DAKO) and normal goat IgG (Santa Cruz Biotechnology) negative control staining was performed and evaluated.

### *In vivo* ubiquitination assay

CNE2-LMP1 and CNE2-vector cells in a 60 mm-diameter dish were cotransfected with the indicated plasmids, including pHA-Ub, pHA-Ub-K48R, pHA-Ub-K63R (lysine at position 48 or 63 was replaced with arginine), pHA-Ub-K48 and pHA-Ub-K63 (all lysines, with the exception of lysines 48 or 63, were replaced with arginine) using Lipofectamine 2000 (Invitrogen). For the detection of GLUT1 ubiquitination, at 36 h post-transfection, the cells were treated with the proteasome inhibitor MG-132 at a final concentration of 20 μM for 6 h and then washed twice with PBS and lysed with ice-cold radioimmunoprecipitation assay (RIPA) buffer (50 mM Tris-HCl [pH 7.5], 150 mM NaCl, 1% NP-40, 0.5% sodium deoxycholate, and 0.1% SDS, supplemented with a protease inhibitor cocktail [Roche]). The supernatants were divided into two aliquots. One aliquot (5%) was prepared for WB. The second aliquot (95%) was sonicated five times to shear the DNA. The soluble lysates were then immunoprecipitated with an anti-hemagglutinin (HA) antibody, followed by three washes with RIPA buffer. HA-tagged proteins were resolved via SDS-PAGE and sequentially blotted with the indicated antibodies.

### Statistical analysis

Parametric statistics were applied for *in vitro* data, Student’s t-tests were used for comparisons between experimental treatment groups and control groups. The relationships among LMP1, GLUT1, CD33 values and the clinical pathological features were tested using Pearson’s chi-square tests. Nonparametric data were analyzed using Mann–Whitney U tests. The Kaplan–Meier method was used to estimate patient survival, and survival differences were estimated using log-rank tests; the cutoff value for each IHC variant was estimated using X-Tile software [67]. The values are presented as the means ± standard errors of the means (SEM). P was considered statistically significant when < 0.05. All data were analyzed using SPSS 18.0 (SPSS, Chicago, IL) and GraphPad Prism 5 software.

## Supporting information

S1 Fig Functional characteristics of the CNE2-LMP1- and CNE2-vector-mediated MDSC populations. The expression levels of suppressive molecules in MDSCs were analyzed by flow cytometry with multiple anti-human mAbs against Arg-1, iNOS, PD-L1 and P-STAT3, as indicated. The gray curve represents autofluorescence as a negative control. Representative histograms are shown.
(TIF)

S2 Fig Association between glycolysis and NPC-associated MDSC differentiation. (**A**) The mRNA levels of glycolytic genes, including GLUT1, HK2, GPI, PFKB2 and PFKB3, in CNE2-LMP1 and TW03-LMP1 cells were decreased after GLUT1 knockdown with a siRNA. (**B**) CNE2 cells induced a higher percentage of tumor-associated HLA-DR^-^CD33^+^CD11b^+^ MDSCs in low pH medium. (**C**) Treatment with si-GLUT1 or 2-DG decreased the expression of suppressive molecules, including Arg-1, iNOS, PD-L1 and P-STAT3, in the NPC-induced MDSC population. (**D**) The percentage of CNE-2-LMP1-induced MDSCs decreased in response to treatment with the anti-metabolic drug DMBG. Representative FACS density plots from 1 of 3 experiments are shown. DMBG, metformin. (**E**). WB showing that GLUT1 expression decreased in CNE2-LMP1 and TW03-LMP1 cells after treatment with DMBG. Data are representative of three independent experiments. DMBG: metformin
(TIF)

S3 Fig LMP1 delays GLUT1 protein degradation by autolysosomes. (**A**) The proteasome inhibitor MG132 increased the half-life of GLUT1 proteins in CNE-2-vector, TW03-vector, CNE2-LMP1 and TW03-LMP1 cells. (**B**) The autophagy inhibitor BafA1 increased GLUT1 expression at different time points in CNE2-vector cells but not in CNE2-LMP1 cells. (**C**) LMP1 binds to p62. NPC-LMP1 and NPC-vector cell lines cultured in 6-well plates were transfected with Flag-tagged p62 (4 μg/well) and then treated with 20 mM MG132 for 6 h prior to harvest. Cell lysates were immunoprecipitated with anti-Flag antibodies and then subjected to WB with an anti-GLUT1 antibody to measure the amount of GLUT1 proteins pulled down by p62 (upper panels). Immunoblotting was performed with anti-Flag and anti-GLUT1 antibodies. β-actin was used as a control. Representative data from 5 independent experiments are shown.
(TIF)

S4 Fig NF-κB inhibition attenuates GLUT1 expression. (A) The level of the GLUT1 mRNA was slightly decreased in CNE2-LMP1 and TW03-LMP1 cells treated with the NF-κB inhibitor BAY. (B) The levels of P-p65, GLUT1, pro-IL-1β and IL-1β were decreased in CNE2-LMP1 and TW03-LMP1 cells treated with the NF-κB inhibitor BAY, but the LMP1, NLRP3 and pro-caspase-1 levels were not affected. Glyceraldehyde 3-phosphate dehydrogenase (GAPDH) was used as a control. Representative data from 3 independent experiments are shown. (C) Results of an ELISA showing that the secretion of IL-1β and IL-6 from CNE2-LMP1 and TW03-LMP1 cells treated with the NF-κB inhibitor BAY was significantly decreased. (D) Statistical analysis of the percentage of CD33^+^CD11b^+^HLA-DR^-^ MDSCs generated from CNE2-LMP1 or TW03-LMP1 cells following the administration of the NF-κB inhibitor BAY. Data are presented as the means ± SEM of representative experiments performed in triplicate. *P < 0.05, **P < 0.01 compared with the control treatment.
(TIF)

S5 Fig Determination of the GLUT1-binding site in LMP1 in NPC. **(A)** Two truncated LMP1 sequences, LMP11-230 (containing the CART1 domain) and LMP1 1–322 (containing CART1, CART3 and CART2 domains), and the full length LMP1 sequence were inserted into plasmid vectors along with Flag tags. (B) The expression of LMP1 and GLUT1 in CNE2 cells transiently transfected with recombinant LMP1 plasmids was detected by immunoblotting. (C) CNE2-LMP1, CNE2-LMP1 1–230 and CNE2-LMP1 1–322 cell lines were treated with CHX for 18 h, proteins were harvested at 0, 3, 6, 12 and 18 h, and the expression of GLUT1 was measured by immunoblotting. Representative data from 5 independent experiments are shown, and GAPDH was included as a control. (D) GLUT1 binding was measured in CNE2-LMP1, CNE2-LMP1 1–230 and CNE2-LMP1 1–322 cell lines using co-IP. Full-length LMP1 and LMP1 1–322 but not LMP1 1–230 were pulled down by GLUT1. Whole-cell lysates (WCLs) were blotted to evaluate the GLUT1 protein levels (lower panels). β-actin expression was used as a protein loading control. The experiment shown is representative of three independent experiments.
(TIF)

S6 Fig Mechanism through which LMP1 regulates GLUT1 expression and its effect on NPC-associated MDSC differentiation. (A) Immunoblot showing that GLUT1 and NLRP3 levels were increased in CNE2 cells that had been transiently transfected with different doses of LMP1 plasmids (μg). (B) CNE2 cells were transfected with hemagglutinin (HA)-tagged ubiquitin (Ub) (4 μg/well), HA-tagged Ub-K48, HA-tagged Ub-K48R or different doses of LMP1 plasmid and then treated with 20 mM MG132 for 6 h prior to harvest. Cell lysates were immunoprecipitated with an anti-HA antibody and then subjected to WB with an anti-GLUT1 antibody to measure the levels of ubiquitinated GLUT1 proteins (upper panels). WCLs were blotted to evaluate the levels of GLUT1 proteins (lower panels). β-actin expression was used as a protein loading control. The experiment shown is representative of three independent experiments. (C) ELISA results showing that the production of cytokines, including IL-1β, IL-6 and GM-CSF, was increased in CNE2 cells that had been transiently transfected with different doses of LMP1 plasmids (μg). (D) The percentage of HLA-DR^-^CD11b^+^CD33^+^ MDSCs was increased in CNE2 cells that had been transiently transfected with different doses of LMP1 plasmids (μg). Data are presented as the means ± SEM of representative experiments performed in triplicate. *P < 0.05, **P < 0.01 compared with the control treatment.
(TIF)

S7 Fig The p62-dependent autolysosomal degradation pathway is involved in LMP1-mediated MDSC induction. (**A**) p62 was knocked down in CNE2-LMP1 and TW03-LMP1 cells. (**B**) The percentage of HLA-DR^-^CD11b^+^CD33^+^ MDSCs generated from CNE2-LMP1 and TW03-LMP1 cells was decreased upon p62 knockdown. Representative data from 1 of 3 independent experiments are shown.
(TIF)

S1 Table Clinical characteristics of the 112 patients with NPC. (PDF)

S2 Table Association of the frequency of high levels of tumor tissue LMP1, GLUT1 and CD33 expression with the clinical parameters of 112 patients with NPC. (PDF)

S3 Table Antibody and reagent information. (PDF)

S4 Table Primers used for qRT-PCR. (PDF)
