# Supplementary figures and images for: LMP1-mediated glycolysis induces myeloid-derived suppressor cell expansion in nasopharyngeal carcinoma
Source: PLoS Pathog. 2017 Jul 21;13(7):e1006503. doi: 10.1371/journal.ppat.1006503 (PMC5540616; doi:10.1371/journal.ppat.1006503)

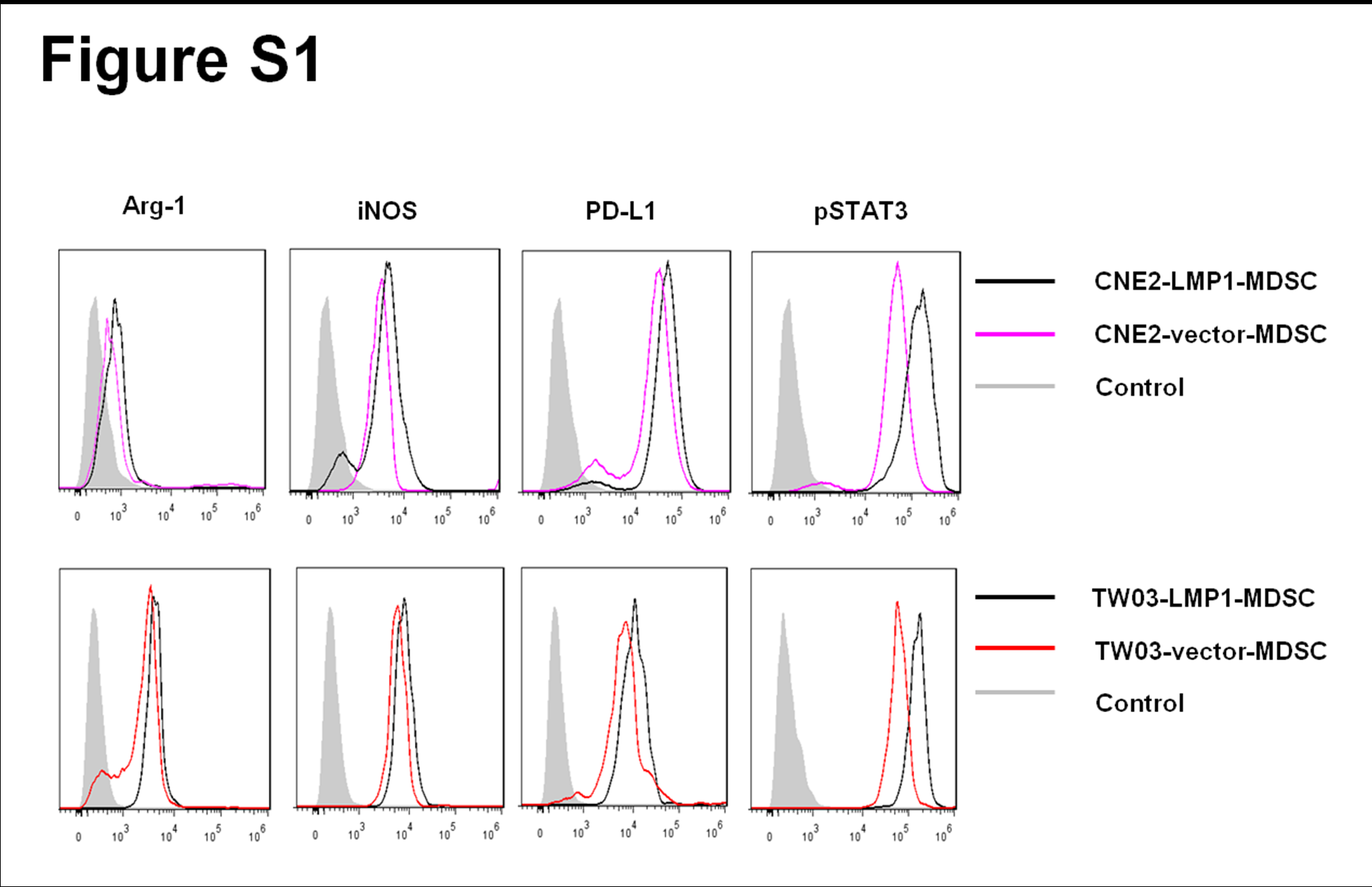

Supplement: S1 Fig — The expression levels of suppressive molecules in MDSCs were analyzed by flow cytometry with multiple anti-human mAbs against Arg-1, iNOS, PD-L1 and P-STAT3, as indicated. The gray curve represents autofluorescence as a negative control. Representative histograms are shown. (TIF) [file ppat.1006503.s001.tif]

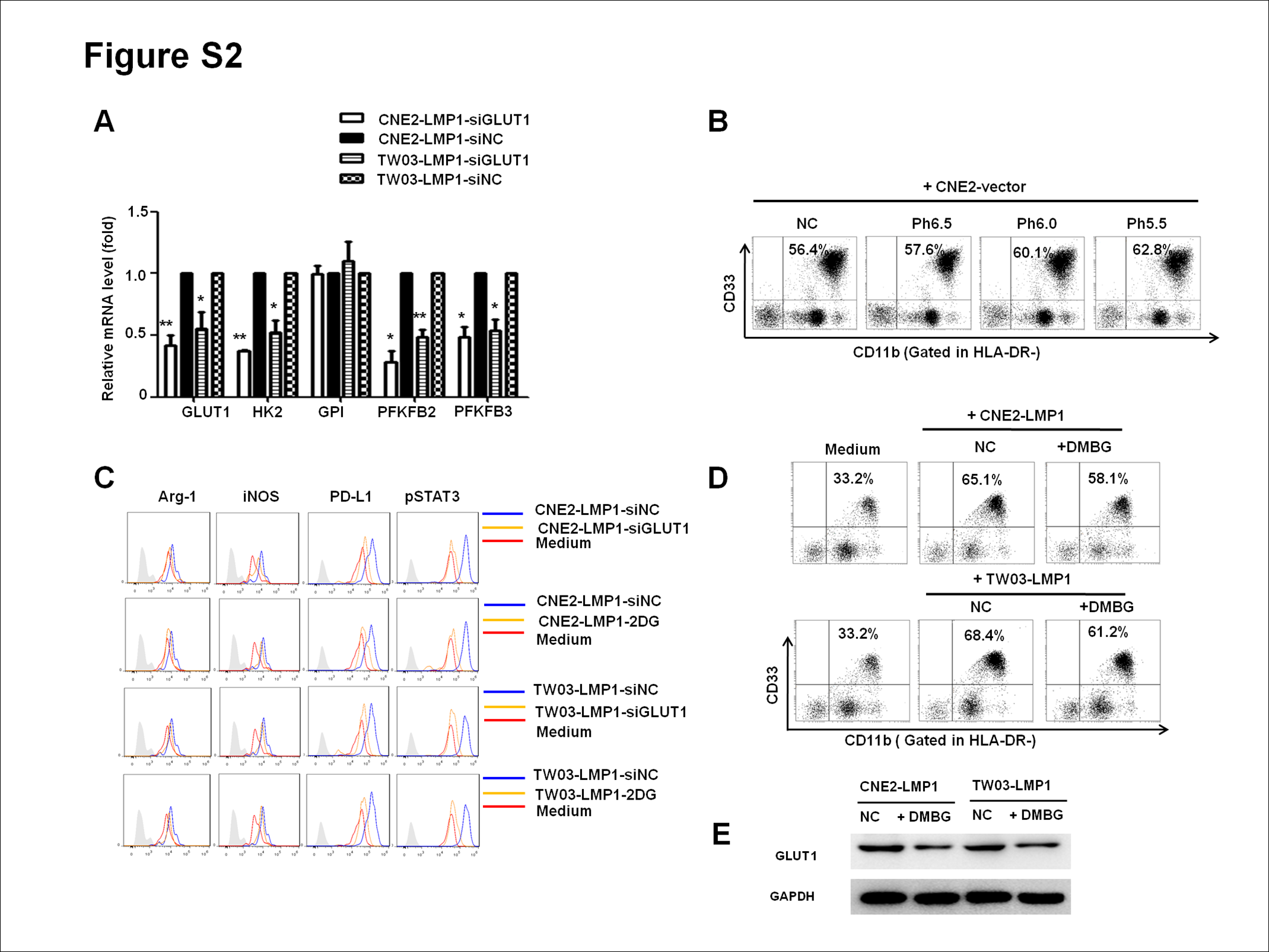

Supplement: S2 Fig — (A) The mRNA levels of glycolytic genes, including GLUT1, HK2, GPI, PFKB2 and PFKB3, in CNE2-LMP1 and TW03-LMP1 cells were decreased after GLUT1 knockdown with a siRNA. (B) CNE2 cells induced a higher percentage of tumor-associated HLA-DR-CD33+CD11b+ MDSCs in low pH medium. (C) Treatment with si-GLUT1 or 2-DG decreased the expression of suppressive molecules, including Arg-1, iNOS, PD-L1 and P-STAT3, in the NPC-induced MDSC population. (D) The percentage of CNE-2-LMP1-induced MDSCs decreased in response to treatment with the anti-metabolic drug DMBG. Representative FACS density plots from 1 of 3 experiments are shown. DMBG, metformin. (E). WB showing that GLUT1 expression decreased in CNE2-LMP1 and TW03-LMP1 cells after treatment with DMBG. Data are representative of three independent experiments. DMBG: metformin (TIF) [file ppat.1006503.s002.tif]

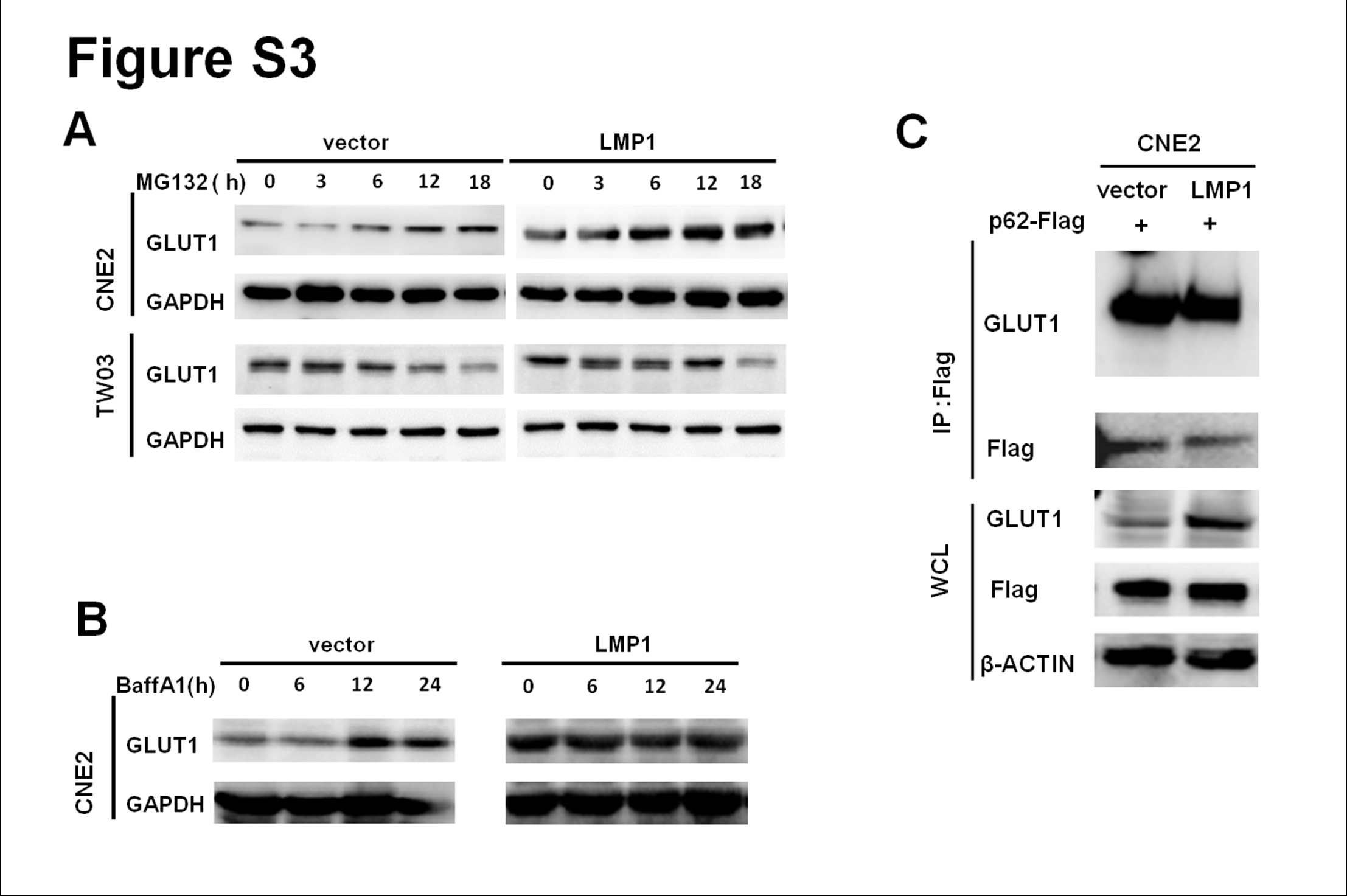

Supplement: S3 Fig — (A) The proteasome inhibitor MG132 increased the half-life of GLUT1 proteins in CNE-2-vector, TW03-vector, CNE2-LMP1 and TW03-LMP1 cells. (B) The autophagy inhibitor BafA1 increased GLUT1 expression at different time points in CNE2-vector cells but not in CNE2-LMP1 cells. (C) LMP1 binds to p62. NPC-LMP1 and NPC-vector cell lines cultured in 6-well plates were transfected with Flag-tagged p62 (4 μg/well) and then treated with 20 mM MG132 for 6 h prior to harvest. Cell lysates were immunoprecipitated with anti-Flag antibodies and then subjected to WB with an anti-GLUT1 antibody to measure the amount of GLUT1 proteins pulled down by p62 (upper panels). Immunoblotting was performed with anti-Flag and anti-GLUT1 antibodies. β-actin was used as a control. Representative data from 5 independent experiments are shown. (TIF) [file ppat.1006503.s003.tif]

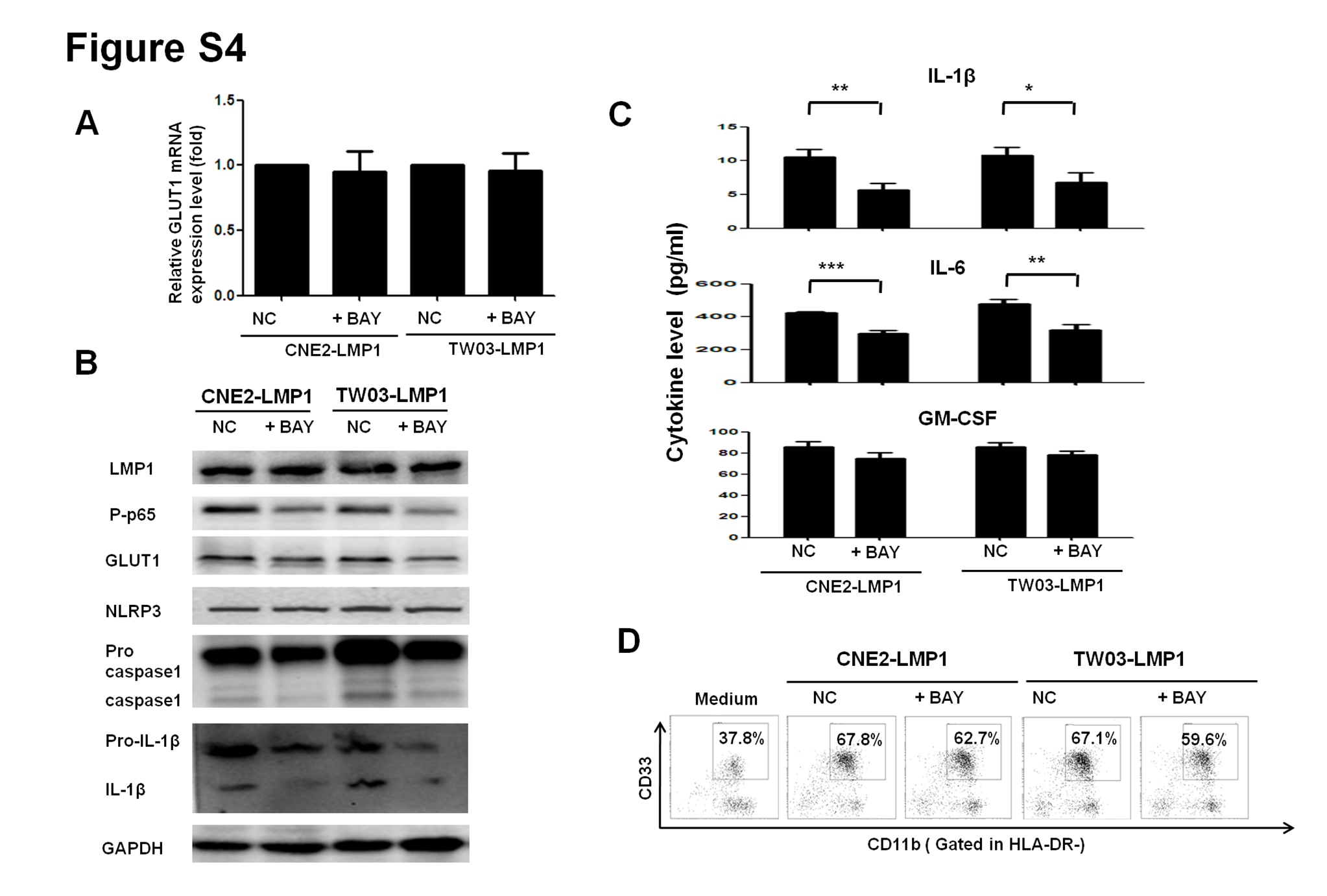

Supplement: S4 Fig — (A) The level of the GLUT1 mRNA was slightly decreased in CNE2-LMP1 and TW03-LMP1 cells treated with the NF-κB inhibitor BAY. (B) The levels of P-p65, GLUT1, pro-IL-1β and IL-1β were decreased in CNE2-LMP1 and TW03-LMP1 cells treated with the NF-κB inhibitor BAY, but the LMP1, NLRP3 and pro-caspase-1 levels were not affected. Glyceraldehyde 3-phosphate dehydrogenase (GAPDH) was used as a control. Representative data from 3 independent experiments are shown. (C) Results of an ELISA showing that the secretion of IL-1β and IL-6 from CNE2-LMP1 and TW03-LMP1 cells treated with the NF-κB inhibitor BAY was significantly decreased. (D) Statistical analysis of the percentage of CD33+CD11b+HLA-DR- MDSCs generated from CNE2-LMP1 or TW03-LMP1 cells following the administration of the NF-κB inhibitor BAY. Data are presented as the means ± SEM of representative experiments performed in triplicate. *P < 0.05, **P < 0.01 compared with the control treatment. (TIF) [file ppat.1006503.s004.tif]

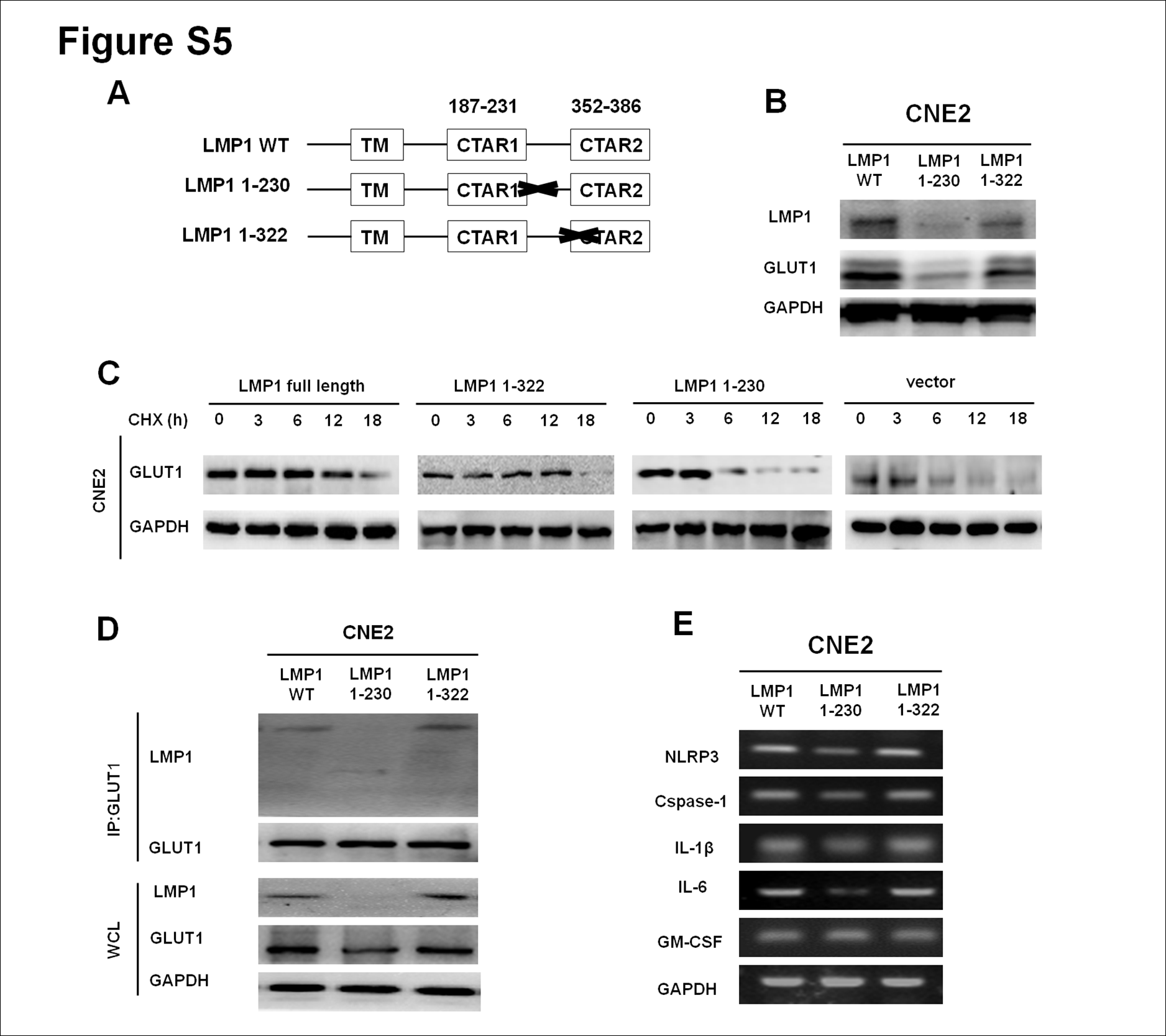

Supplement: S5 Fig — (A) Two truncated LMP1 sequences, LMP11-230 (containing the CART1 domain) and LMP1 1–322 (containing CART1, CART3 and CART2 domains), and the full length LMP1 sequence were inserted into plasmid vectors along with Flag tags. (B) The expression of LMP1 and GLUT1 in CNE2 cells transiently transfected with recombinant LMP1 plasmids was detected by immunoblotting. (C) CNE2-LMP1, CNE2-LMP1 1–230 and CNE2-LMP1 1–322 cell lines were treated with CHX for 18 h, proteins were harvested at 0, 3, 6, 12 and 18 h, and the expression of GLUT1 was measured by immunoblotting. Representative data from 5 independent experiments are shown, and GAPDH was included as a control. (D) GLUT1 binding was measured in CNE2-LMP1, CNE2-LMP1 1–230 and CNE2-LMP1 1–322 cell lines using co-IP. Full-length LMP1 and LMP1 1–322 but not LMP1 1–230 were pulled down by GLUT1. Whole-cell lysates (WCLs) were blotted to evaluate the GLUT1 protein levels (lower panels). β-actin expression was used as a protein loading control. The experiment shown is representative of three independent experiments. (TIF) [file ppat.1006503.s005.tif]

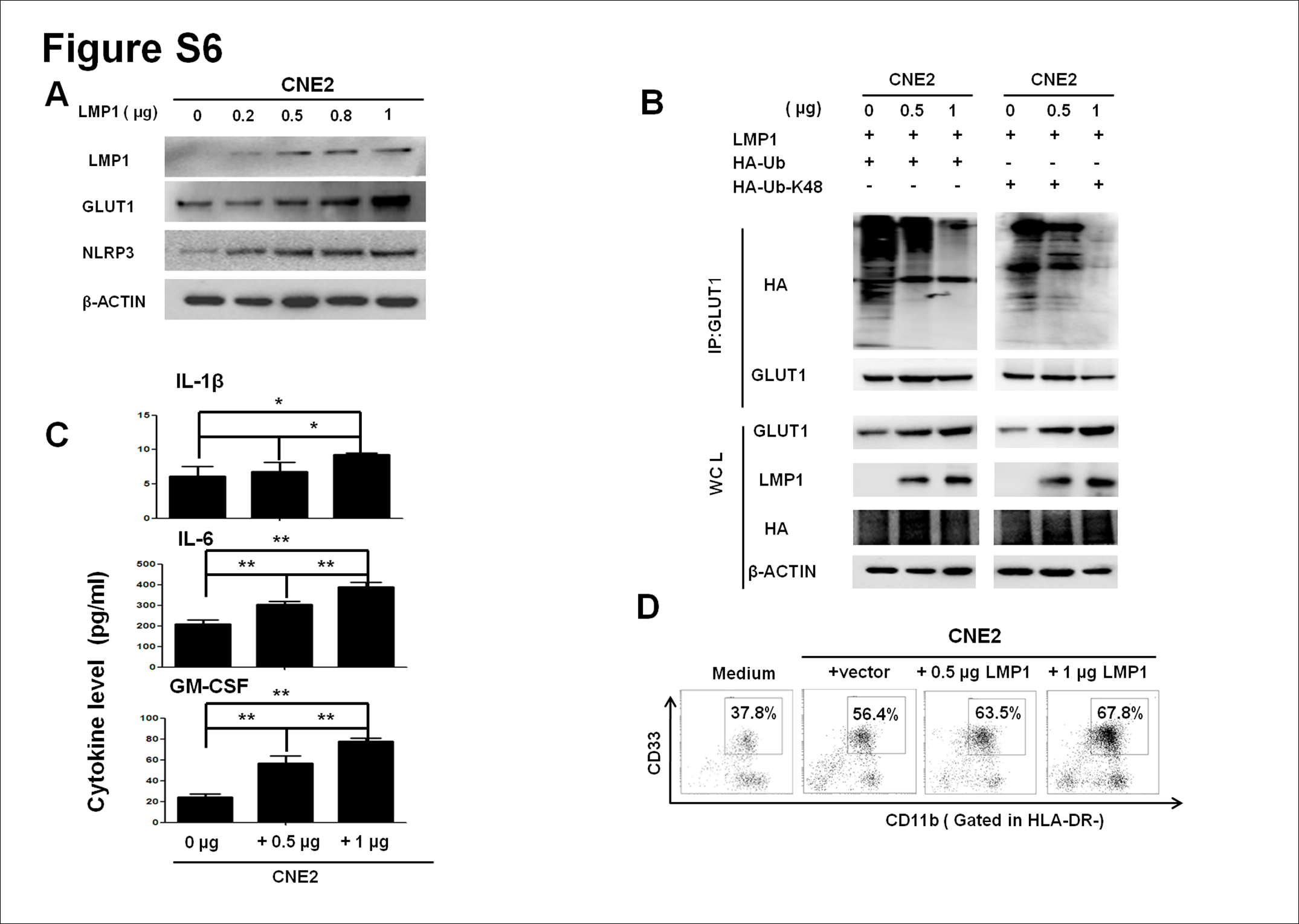

Supplement: S6 Fig — (A) Immunoblot showing that GLUT1 and NLRP3 levels were increased in CNE2 cells that had been transiently transfected with different doses of LMP1 plasmids (μg). (B) CNE2 cells were transfected with hemagglutinin (HA)-tagged ubiquitin (Ub) (4 μg/well), HA-tagged Ub-K48, HA-tagged Ub-K48R or different doses of LMP1 plasmid and then treated with 20 mM MG132 for 6 h prior to harvest. Cell lysates were immunoprecipitated with an anti-HA antibody and then subjected to WB with an anti-GLUT1 antibody to measure the levels of ubiquitinated GLUT1 proteins (upper panels). WCLs were blotted to evaluate the levels of GLUT1 proteins (lower panels). β-actin expression was used as a protein loading control. The experiment shown is representative of three independent experiments. (C) ELISA results showing that the production of cytokines, including IL-1β, IL-6 and GM-CSF, was increased in CNE2 cells that had been transiently transfected with different doses of LMP1 plasmids (μg). (D) The percentage of HLA-DR-CD11b+CD33+ MDSCs was increased in CNE2 cells that had been transiently transfected with different doses of LMP1 plasmids (μg). Data are presented as the means ± SEM of representative experiments performed in triplicate. *P < 0.05, **P < 0.01 compared with the control treatment. (TIF) [file ppat.1006503.s006.tif]

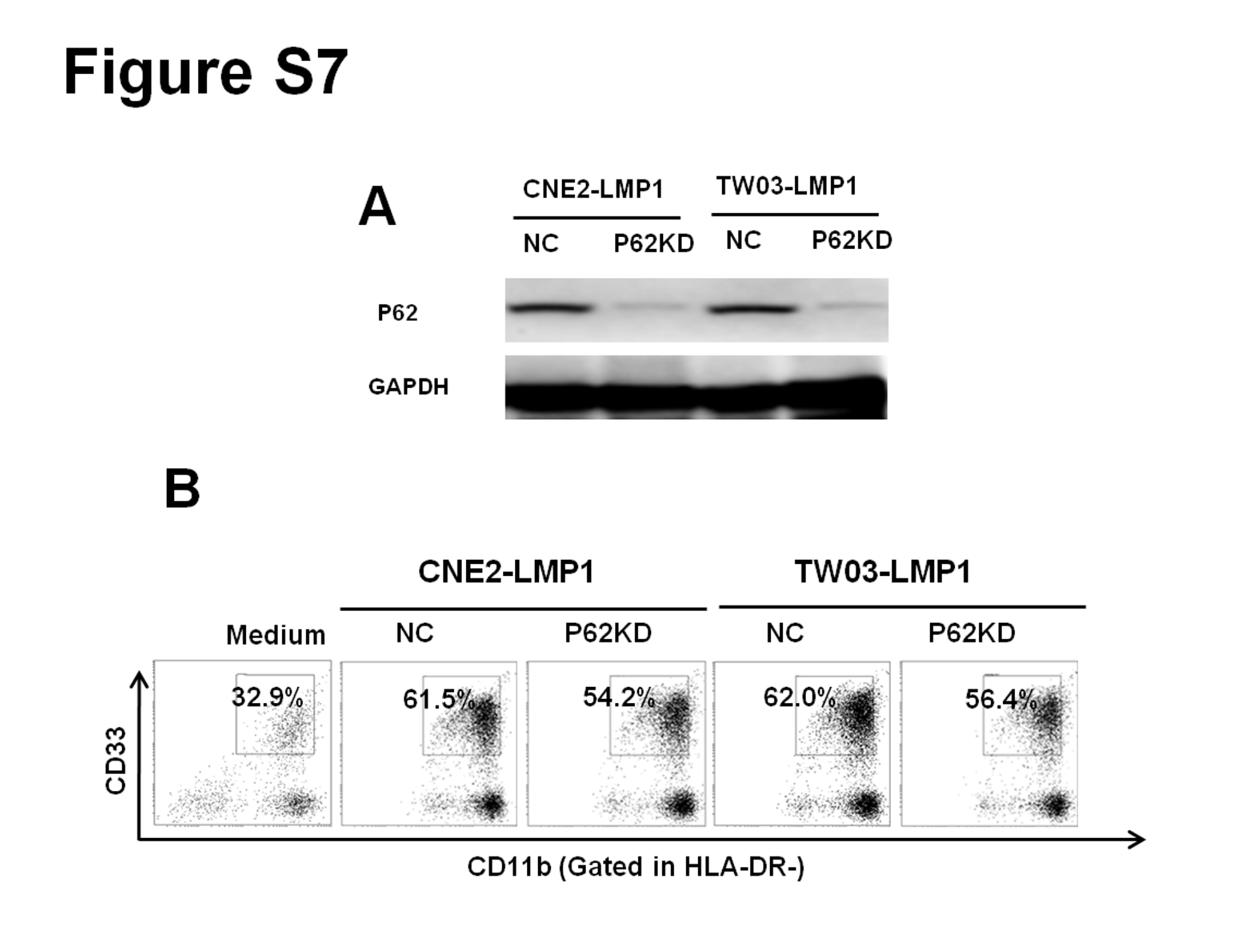

Supplement: S7 Fig — (A) p62 was knocked down in CNE2-LMP1 and TW03-LMP1 cells. (B) The percentage of HLA-DR-CD11b+CD33+ MDSCs generated from CNE2-LMP1 and TW03-LMP1 cells was decreased upon p62 knockdown. Representative data from 1 of 3 independent experiments are shown. (TIF) [file ppat.1006503.s007.tif]
