# Supplementary material for: LMP1-mediated glycolysis induces myeloid-derived suppressor cell expansion in nasopharyngeal carcinoma
Source: PLoS Pathog. 2017 Jul 21;13(7):e1006503. doi: 10.1371/journal.ppat.1006503 (PMC5540616; doi:10.1371/journal.ppat.1006503)
Supplement: S1 Table — (PDF) [file ppat.1006503.s008.pdf]

Table S1. The clinical characteristics of 112 patients with NPC.

Characteristic	No. (%) of patients
**Total cases**	112 (100%)
**Age**	
Mean	45.95
Range	22-75
**Gender**	
Male	92 (82.1%)
Female	20(17.9%)
**Tumor (T) status**	
T1	2 (1.8%)
T2	26 (23.2%)
T3	64 (57.1%)
T4a-b	20 (17.9%)
**Lymph node (N) status**	
N0	16 (14.3%)
N1a-b	50 (44.6%)
N2	36 (32.1%)
N3	10 (8.9%)
**Distant metastasis (M) status**	
M0	111(99.1%)
M1	1 (0.9%)
**TNM stage**	
I	2 (1.8%)
IIa-b	20 (17.9%)
III	61 (54.5%)
IV	29 (25.9%)
**Relapse**	
No	103 (91.96%)
Yes	9 (8.04%)
**Death**	
No	109(97.4%)
Yes	3 (2.6%)
