# Supplementary material for: LMP1-mediated glycolysis induces myeloid-derived suppressor cell expansion in nasopharyngeal carcinoma
Source: PLoS Pathog. 2017 Jul 21;13(7):e1006503. doi: 10.1371/journal.ppat.1006503 (PMC5540616; doi:10.1371/journal.ppat.1006503)
Supplement: S2 Table — (PDF) [file ppat.1006503.s009.pdf]

Table S2. Antibody information.

Name	Lot number (clone number)	Brand	Production area
**For flow cytometric analysis**			
Mouse anti-human CD8	12-0088-42 (RPA-T8)	eBioscience	San Diego, CA, USA
Mouse anti-human ARG1	ABPT0315061(/)	Bio-Techne	Minneapolis, MN, USA
Mouse anti-human PD-L1	11-9969-42 (MIH4)	eBioscience	San Diego, CA, USA
Mouse anti-human P-STAT3	17-9033-41 (LUVNKLA)	eBioscience	San Diego, CA, USA
Mouse anti-human iNOS	K1413(/)	Santa Cruz Biotechnology	Delaware Ave Santa Cruz, CA, USA
Mouse anti-human CD11b	25-0118-42 (ICRF44)	eBioscience	San Diego, CA, USA
Mouse anti-human CD4	555346 (RPA-T4)	BD Bioscience	San Jose, CA, USA
Mouse anti-human CD33	45-0338-42 (WM53)	eBioscience	San Diego, CA, USA
Mouse anti-human HLA-DR	12-9956-42 (LN3)	eBioscience	San Diego, CA, USA
**For Western blot analyses**			
Mouse anti-human LMP1	GM089729 (CS1-4)	Gene Tech	Shanghai, ZY, China
Rabbit anti-human GLUT1	NB110-39113 (/)	Novus Biologicals	Littleton, CO, USA
Rabbit anti-human NLRP3	19771-1-AP (/)	Proteintech	Rosemont, IL, USA
Rabbit anti-human IL-1β	16806-1-AP (/)	Proteintech	Rosemont, IL, USA
Rabbit anti-human Caspase-1	2225 (/)	Cell Signaling Technology	Danvers, MA, China
Mouse anti-human GAPDH	AG019 (6C5)	Beyotime	Guangzhou, GD, China
Mouse anti-human β-actin	AA128 (AC-74)	Beyotime	Guangzhou, GD, China
**For immunohistochemistry**			
Mouse anti-human LMP1	GM089729 (CS1-4)	Gene Tech	Shanghai, ZY, China
Rabbit anti-human GLUT1	NB110-39113 (SLC2A1)	Novus Biologicals	Littleton, CO, USA
