# Supplementary material for: LMP1-mediated glycolysis induces myeloid-derived suppressor cell expansion in nasopharyngeal carcinoma
Source: PLoS Pathog. 2017 Jul 21;13(7):e1006503. doi: 10.1371/journal.ppat.1006503 (PMC5540616; doi:10.1371/journal.ppat.1006503)
Supplement: S3 Table — (PDF) [file ppat.1006503.s010.pdf]

Table S3. Primers used for qRT-PCR.

Gene	Forward	Reverse
GLUT-1	5ˈ- ATTGGCTCCGGTATCGTCAAC-3ˈ	5ˈ-GCTCAGATAGGACATCCAGGGTA-3ˈ
HK2	5ˈ-TGCCACCAGACTAAACTAGACG-3ˈ	5ˈ-CCCGTGCCCACAATGAGAC-3ˈ
GPI	5ˈ-CCGCGTCTGGTATGTCTCC-3ˈ	5ˈ-CCTGGGTAGTAAAGGTCTTGGA-3ˈ
G6PD	5ˈ-CGAGGCCGTCACCAAGAAC-3ˈ	5ˈ-GTAGTGGTCGATGCGGTAGA-3ˈ
PFKFB1	5ˈ- GGTGCCCGTGTCTTCTTTGT-3ˈ	5ˈ- AAGCATCATCGAAACGCTCTC-3ˈ
PFKFB2	5ˈ- AGTCCTACGACTTCTTTCGGC-3ˈ	5ˈ- TCTCCTCAGTGAGATACGCCT-3ˈ
PFKFB3	5ˈ- ATTGCGGTTTTCGATGCCAC-3ˈ	5ˈ- GCCACAACTGTAGGGTCGT-3ˈ
PFKFB4	5ˈ- TCCCCACGGGAATTGACAC-3ˈ	5ˈ- GGGCACACCAATCCAGTTCA-3ˈ
ALDOA	5ˈ- ATGCCCTACCAATATCCAGCA-3ˈ	5ˈ- GCTCCCAGTGGACTCATCTG-3ˈ
PGK1	5ˈ- GACCTAATGTCCAAAGCTGAGAA-3ˈ	5ˈ- CAGCAGGTATGCCAGAAGCC-3ˈ
PKM 2	5ˈ- ATGTCGAAGCCCCATAGTGAA-3ˈ	5ˈ- TGGGTGGTGAATCAATGTCCA-3ˈ
PDK1	5ˈ- GAGAGCCACTATGGAACACCA-3ˈ	5ˈ- GGAGGTCTCAACACGAGGT-3ˈ
LDHA	5ˈ- TCTCTGTAGCAGATTTGGCAGA-3ˈ	5ˈ-AAGACATCATCCTTTATTCCGTAA-3ˈ
NLRP3	5ˈ-CGTGAGTCCCATTAAGATGGAGT-3ˈ	5ˈ-CCCGACAGTGGATATAGAACAGA-3ˈ
CASpase-1	5ˈ-GTTTCTTGGAGACATCCC -3ˈ	5ˈ-TAATGTCCTGGGAAGAGG -3ˈ
ASC	5ˈ-TGGATGCTCTGTACGGGAAG -3ˈ	5ˈ- CCAGGCTGGTGTGAAACTGAA-3ˈ
IL-1β	5ˈ-ATGATGGCTTATTACAGTGGCAA-3ˈ	5ˈ-GTCGGAGATTCGTAGCTGGA -3ˈ
COX-2	5ˈ-CTGGCGCTCAGCCATACAG-3ˈ	5ˈ- CGCACTTATACTGGTCAAATCCC-3ˈ
ARG1	5ˈ-TGGACAGACTAGGAATTGGCA-3ˈ	5ˈ-CCAGTCCGTCAACATCAAAACT-3ˈ
iNOS	5ˈ-TTCAGTATCACAACCTCAGCAAG-3ˈ	5ˈ-TGGACCTGCAAGTTAAAATCCC -3ˈ
ACTIN	5ˈ- AGAGCTACGAGCTGCCTGAC-3ˈ	5ˈ-TCTGGCTTGTTCCTCACTACT-3ˈ
GAPDH	5ˈ-CGAGATCCCTCCAAAATCAAGTGGGG-3ˈ	5ˈ-ACACGTTGGCAGTGGGGACAC-3ˈ
