# Supplementary material for: LMP1-mediated glycolysis induces myeloid-derived suppressor cell expansion in nasopharyngeal carcinoma
Source: PLoS Pathog. 2017 Jul 21;13(7):e1006503. doi: 10.1371/journal.ppat.1006503 (PMC5540616; doi:10.1371/journal.ppat.1006503)
Supplement: S4 Table — (PDF) [file ppat.1006503.s011.pdf]

Table S4. The clinical characteristics of 112patients with NPC

Variables	Univariate analysis		Multivariate analysis	
	HR (95% CI)	*P*-value	HR (95% CI)	*P*-value
**Disease free survival**				
**Age** (<46/≥46)	1.407(0.377-5.253)	0.611		
**Gender** (Female/Male)	0.644 (0.810-5.152)	0.678		
**Clinical Stage** (Ⅰ--Ⅱ/III--Ⅳ)	1.985(0.731-5.395)	0.179		
**TNM Stage** (Ⅰ--Ⅱ/III--Ⅳ)	30.493 (0.041-22489.933)	0.310		
**T Stage** (T1-T2/T3-T4)	34.534 (0.077-15450.152)	0.225		
**N Stage** (N0-N1/N2-N3)	27.107 (0.016-47362.027)	0.386		
**Lmp1 Score**	9.028 (1.129-72.206)	**0.038***	8.057(0.976-66.538)	0.053
**CD33 Score**	4.245 (0.880-20.481)	**0.072***	4.506(0.893-22.745)	0.068
**DNA copy**	7.991 (0.976-65.402)	**0.053***	9.691(1.167-80.458)	**0.035***
**EA**	0.473(0.150-1.485)	0.199		
**VCA**	3.413(0.497-23.423)	0.212		
